# Salivary oxytocin responses to infant stimuli vary by EPDS scores among postpartum Japanese mothers without clinically diagnosed postpartum depression

**DOI:** 10.3389/fendo.2025.1689899

**Published:** 2025-12-17

**Authors:** Kana Minami, Haruhiro Higashida, Shigeru Yokoyama, Takahiro Tsuji, Naomi Kagami, Chiharu Tsuji

**Affiliations:** 1 Research Center for Child Mental Development, Kanazawa University, Kanazawa, Japan; 2 Department of Health Development Nursing, Institute of Medical, Pharmaceutical and Health Sciences, Kanazawa University, Kanazawa, Japan; 3 Department of Socioneurosciences, United Graduate School of Child Development, Osaka University, Kanazawa University, Hamamatsu University School of Medicine, Chiba University, and University of Fukui, Kanazawa, Japan; 4 Department of Ophthalmology, Faculty of Medical Sciences, University of Fukui, Fukui, Japan

**Keywords:** postpartum depression, oxytocin, EPDS, mother, saliva

## Abstract

Postpartum depression (PPD) significantly impacts both mothers and children, so its early detection is crucial to mitigate these effects. The Edinburgh Postnatal Depression Scale (EPDS) is a widely used screening tool for identifying individuals at risk of PPD. However, PPD symptoms often emerge gradually, and subtle changes in maternal well-being within the low-risk EPDS range may be overlooked. Oxytocin (OT), a neuropeptide important for social functioning and maternal behaviors, may offer deeper insights into the progression of PPD. This exploratory cross-sectional study examined the association between EPDS scores and salivary OT responses to infant-related stimuli in postpartum Japanese mothers without PPD diagnosis. We hypothesized that OT responses would differ according to mothers’ EPDS scores, with higher scores associated with blunted OT reactivity. OT responses were assessed within one year postpartum under breastfeeding, interaction, or video tests. Mothers with EPDS <5 showed increased OT responses, whereas those with ≥5 showed diminished responses. The difference in OT responses observed below the EPDS screening cutoff may suggest early biological sensitivity associated with PPD vulnerability. Although the factors determining who develops PPD remain unclear, our findings may highlight the potential value of integrating OT response assessments with EPDS screening to improve early detection. Further, these findings suggest that OT dynamics may serve as a biological indicator of subtle emotional changes during the postpartum period.

## 1 Introduction

Postpartum depression (PPD) is a significant mental health disorder affecting 10%–20% of mothers. It is characterized by symptoms such as depressed mood, anhedonia, sleep disturbances, low self-esteem, and suicidal thoughts (1). Unlike transient mood swings such as “maternity blues,” PPD is often prolonged and can severely impact maternal nurturing behaviors, including attachment formation, interest in the child, and willingness to breastfeed. However, with appropriate treatment, ~70% of patients with PPD recover within the first year (1). Nevertheless, the increasing suicide rate among women perinatally has become a pressing issue, to which PPD makes a major contribution (2). Furthermore, PPD not only affects mothers, but also has long-term adverse effects on their children (3–6). There is thus an urgent need to detect PPD at an early stage to mitigate its adverse effects on both mother and child, and early biological or behavioral indicators may help identify risk before PPD appears.

PPD is often screened using the Edinburgh Postnatal Depression Scale (EPDS), a globally used, cost-effective questionnaire (7). The EPDS consists of a 10-item self-reported questionnaire (8). Each question corresponds to a common symptom of depression, with higher scores indicating a more severely depressed state. While the cutoff score varies by country, it is generally highly sensitive and specific (9); a score of ≥12/13 suggests possible depression and that further clinical evaluation is recommended (10). In Japan, it has been reported that the optimal cut-off score for the EPDS is 9, with a sensitivity of 75-82% and specificity of 93-95% (11, 12). Despite the value of using a threshold of this kind, it is important to note that mental distress does not suddenly emerge when a score exceeds this value. Instead, subtle, gradual changes often precede the onset of depressive symptoms. Even mothers with scores below the cutoff may experience subclinical levels of distress that can affect their daily lives and overall well-being. Unfortunately, these subclinical situations are often overlooked, leaving mothers without the support and intervention they may need. While many studies have focused on the differences between those with and without a clinical diagnosis of PPD, few have examined the progression into a depressed state.

OT is a neuropeptide hormone produced in the hypothalamus and released into the circulation through the neurohypophysis system, which plays a crucial role in maternal functions (13). It is released in the periphery to promote parturition through uterine contraction and to induce the milk ejection reflex during breastfeeding (13). OT also promotes various affiliative and prosocial behaviors in humans and facilitates maternal behaviors such as bonding and caregiving (13–16). Like many other placental hormones, plasma OT levels generally rise toward the end of pregnancy and decrease within 3–5 days after delivery (17, 18). A rapid decrease in the plasma OT level after delivery is potentially associated with increased maternal susceptibility to mood disturbances. Consequently, numerous studies have investigated OT levels in relation to postpartum depressive symptoms during the pre- and postpartum periods. These studies found an association between the plasma OT levels in late pregnancy and/or postpartum and the EPDS scores (19, 20). Skrundz et al. found that lower OT during midpregnancy was linked with an EPDS score of >10 at 2 weeks postpartum, representing a risk for PPD (21). Moreover, Cox et al. reported that breastfeeding women with depressive symptoms, defined by EPDS ≥10 and/or State-Trait Anxiety Inventory (STAI) test score ≥34, had lower overall plasma OT levels during breastfeeding, compared with asymptomatic breastfeeding women (22). Therefore, lower OT levels have often been linked to depressive symptoms (19). However, no studies have specifically examined OT levels in mothers scoring below the EPDS cutoff.

Meanwhile, emerging evidence suggests that disruptions in the OT system are well associated with impairments in social functioning, underscored by the link between OT system imbalances and socioemotional dysfunctions in psychiatric disorders (13, 21–25). Indeed, there are studies on motherhood that focus on the correlation of the OT system with specific aspects of social functioning (26–29). For instance, Strathearn et al. examined the relationship between the intensity of a mother’s attachment and her OT response to her infant (30). They found that “secure” mothers exhibited an increased OT response during such interactions, whereas no increase was found in “insecure” mothers. This suggests that detecting OT system dysfunction in such mothers may be particularly relevant within social contexts. Nonetheless, no studies have specifically examined whether maternal depressive symptoms, particularly among mothers scoring below the EPDS cutoff, are associated with altered OT responses to infant-related social cues. Understanding this relationship is important, as social cue-elicited OT responses may reveal subtle variations in maternal emotional state that may not be detected when relying solely on EPDS cutoff scores to separate “high-risk” and “low-risk” mothers.

To address this gap, we investigated the association between maternal mental status and OT response to infant-related stimuli in postpartum Japanese mothers without a clinical diagnosis of PPD. Our prior observations in postpartum mothers scoring ≤3 on the EPDS showed that OT levels increase rapidly in response to infant-related stimuli (31). Informed by these prior observations confirming OT reactivity to infant stimuli, we hypothesized that even among mothers without a clinical diagnosis of PPD, OT response patterns to infant stimuli would differ according to psychological state, such that higher EPDS scores are associated with relatively more blunted OT responses.

## 2 Materials and methods

### 2.1 Study design and setting

This was an exploratory cross-sectional study conducted among postpartum mothers in Japan without a clinical diagnosis of PPD. The study investigated associations between maternal psychological status and OT responses to infant-related stimuli, including breastfeeding, direct mother–infant interaction, and infant video viewing. Data collection took place between 2018–2022. For the breastfeeding test, sampling occurred either in a university multipurpose Japanese-style room or in participants’ homes, depending on each participant’s preference. The interaction and video tests were conducted in participants’ homes.

### 2.2 Participants

Eligible participants were mothers at 1–12 months postpartum who had reached full-term gestation without maternal or infant abnormalities and were currently breastfeeding. They were physically healthy and capable of completing the study procedures, with no diagnosis of psychiatric disorders or history of psychiatric medication use.

**Figure 1:**
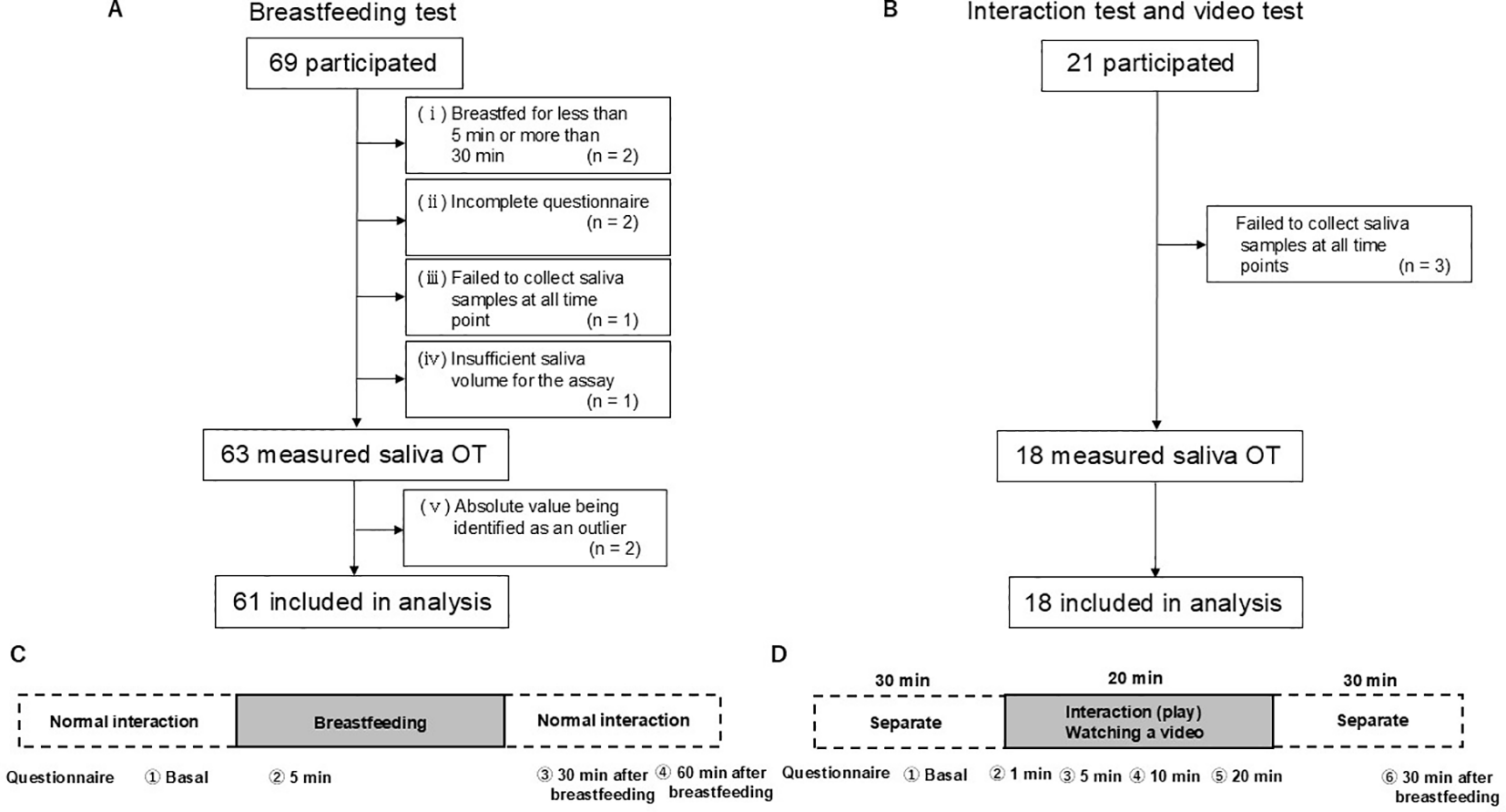
Experimental timeline for the collection of saliva samples and participant flow throughout the study. **(A, B)** The diagram displays the number of participants who were enrolled, excluded, and used for generating the figure. **(C)** Samples were collected 10 min before (basal) and 5 min after the initiation of breastfeeding. The post feeding samples were collected 30 and 60 min after feeding ended. **(D)** Saliva samples were collected at the following timepoints: before the interaction or video test (timepoint 1), or at 1 (timepoint 2), 5 (timepoint 3), 10 (timepoint 4), and 20 min (timepoint 5) after the start of the interaction or video watching. The posttest sample was collected 30 min after the interaction or watching the video (timepoint 6).

Three types of experimental tests were conducted: a breastfeeding test (1–12 months postpartum) and interaction and video tests (6–7 months postpartum), (**Figure 1**). For the interaction and video tests, only mothers who could participate in both tests were enrolled following procedures established in our previous study (31).

Recruitment was conducted as follows: For the recruitment of breastfeeding test, pediatricians identified eligible mothers during routine infant health checkups. Each received written and verbal explanations of the study from a pediatrician and provided verbal consent. At that time, mothers chose whether to provide both saliva and blood samples or only saliva samples. Those who selected saliva-only sampling received written instructions, a consent form, a questionnaire, and a saliva collection kit from the pediatrician during the checkup, and later conducted the experiment at home. Mothers who opted for both saliva and blood sampling contacted the researcher after the checkup to schedule their session and subsequently visited the university for the experiment. Separately, for the interaction and video tests, recruitment posters were displayed on clinic bulletin boards, and interested mothers approached their pediatricians. The pediatrician determined the eligibility and referred mothers to the research team. Researchers then explained the study by phone or e-mail. Participants who agreed to join both tests made appointments for home visits. During the visit, participants received written and verbal explanations of the study and provided written informed consent. They then chose to provide either saliva only or both saliva and blood samples.

All eligible mothers were enrolled without any exclusions. After data collection, exclusions were applied according to predefined criteria (see Section 2.11). A total of 69 and 21 mothers participated in the breastfeeding test and the interaction/video test, respectively. After applying the exclusion criteria for the breastfeeding and interaction/video tests, data from 61 and 18 mothers were included in the final analysis of OT level, respectively, as illustrated in **Figures 1A, B**. All participants in this study were newly recruited and had not participated in any of our previous studies. Follow-up data were not collected, as participation consisted of a single testing session per protocol.

### 2.3 Ethics statement

This study was approved by the Kanazawa University Medical Ethics Review Committee (approval number: 3096-4). It was conducted in accordance with the Ethical Guidelines for Clinical Studies of the Ministry of Health, Labor and Welfare of Japan and the principles of the Declaration of Helsinki. Written informed consent was obtained from all participants.

### 2.4 Questionnaires

Participants completed the Japanese versions of the EPDS, State–Trait Anxiety Inventory (STAI), Stress Response Scale (SRS-18), and Mother-to-Infant Bonding Scale (MIBS-J). In addition, a structured demographic questionnaire and a physiological/lifestyle questionnaire were administered to assess factors relevant to hormonal measurements. Demographic variables included maternal age, educational background, obstetric history, infant birth weight, family type, employment status, and mode of delivery (vaginal or cesarean). Physiological/lifestyle variables included smoking habits, medication use, menstrual status, time since last meal, time since last breastfeeding, and breastfeeding intensity. All questionnaires were completed on the day of the experiment. Demographic and breastfeeding intensity data are summarized in **Tables 1**–**6**. These variables were evaluated to describe the sample population rather than to assess heterogeneity or to serve as confounders in the analyses; therefore, no statistical adjustments were applied.

**Table 1:** Demographic and obstetric characteristics in breastfeeding test (n = 61).

Variable	Category	n (%)	Mean ± S.D.	Min.	n=61 Max.
Mother Age (year)			33.1 ± 4.2	25	46
Obstetric History	Primipara	15 (24.6)			
	Multipara	46 (75.4)			
Delivery	Vaginal delivery	40 (65.6)			
	Caesarian section	21 (34.4)			
Infant Age	1 month	12 (19.7)			
	3 months	17 (27.9)			
	6-7 months	24 (39.3)			
	12 months	8 (13.1)			
Infant Birth weight			3014.3 ± 353.8	2192	3880
Education	4-year college guraduate	23 (37.7)			
	Guraduate 4 years college	36 (59.0)			
	Postgraduate	2 (3.3)			
Family Type	Nuclear family	53 (86.9)			
	Extended family	8 (13.1)			
Employment Situation	Working	5 ( 8.2 )			
	Maternity leave	49 (80.3)			
	Housewife	7 (11.5)			
Menstruation	Postpartum amenorrhea	47 (77.0)			
	Restart	14 (23.0)			
EPDS (0-30)			3.7 ± 3.0	0	12
STAI (state-anxiety) (20-80)			38.9 ± 9.0	24	57
(trait-anxiety) (20-80)			33.7 ± 7.6	22	50
SRS-18 (0-54)			8.3 ± 7.6	0	30
MIBS-J (30-0)			1.5 ± 1.7	0	9

**Table 2:** Demographic and obstetric characteristics in breastfeeding test classified by EPDS score.

Variable	Category	L-group (EPDS < 5) n = 44				H-group (EPDS ≥ 5) n=17				Statistics
		n (%)	Mean ± SD^†^	Min.	Max.	n (%)	Mean ± SD^†^	Min.	Max.	P-value
Mother Age (year)			33.1 ± 4.1	25	46		33.2 ± 4.6	27	40	0.92
Obstetric History	Primipara	12 (27.3)				3 (17.8)				
	Multipara	32 (72.7)				14 (82.4)				
Delivery	Vaginal delivery	30 (68.2)				10 (58.8)				
	Caesarian section	14 (31.8)				7 (41.2)				
Postpartum Periods	1 month	10 (22.7)				2 (11.8)				
	3 months	13 (29.5)				4 (23.5)				
	6-7 months	16 (34.1)				8 (47.1)				
	12 months	5 (11.4)				3 (17.6)				
Infant birth weight			3,040 ± 367.8	2,192	3,880		2,949 ± 315.4	2,336	3,462	0.39
Education	<4 years college graduate	19 (43.2)				4 (23.5)				
	Graduate 4 years college	23 (52.3)				13 (76.5)				
	Postgraduate	2 (4.5)				0 (0.0)				
Family Type	Nuclear family	39 (88.6)				14 (82.4)				
	Extended family	5 (11.4)								
Employment Situation	Working	3 (6.8)				2 (11.8)				
	Maternity leave	38 (86.4)				11 (64.7)				
	Housewife	3 (6.8)				4 (23.5)				
Menstruation	Postpartum amenorrhea	34 (77.3)				13 (76.5)				
	Restart	10 (22.7)				4 (23.5)				
EPDS (0-30)			2.1 ± 1.3	0	4		7.8 ± 1.9	5	12	< 0.001^***^
STAI state (20-80)			36.2 ± 7.4	24	52		45.8 ± 9.2	24	57	< 0.001^***^
STAI trait (20-80)			32.4 ± 6.6	22	50		37.1 ± 9.1	24	49	0.14
SRS-18 (0-54)			6.8 ± 6.6	0	26		12.4 ± 8.7	0	30	0.02^*^
MIBS-J (30-0)			1.5 ± 1.8	0	9		1.5 ± 1.5	0	5	0.82

^†^SD, standard deviation, Significance level is shown p-value *p < 0.05, ***p < 0.001, (Mann-Whitney's U).

**Table 3:** Breastfeeding intensity in breastfeeding test.

Breastfeeding methods	L–group (EPDS < 5) n = 44				H–group (EPDS ≥ 5) n = 17			
	1 month n = 10	3 months n = 13	6-7 months n = 16	12 months n = 5	1 month n = 2	3 months n = 4	6-7 months n = 8	12 months n = 3
1. Exclusive breastmilk	7 (70.0)	10 (76.9)	―	―	―	―	―	―
2. More breastmilk than formula (baby food)	3 (30.0)	2 (15.3)	10 (62.5)	―	―	2 (50.0)	2 (25.0)	―
3. About the same amount of breastmilk and formula (baby food)	―	1 (7.7)	6 (37.5)	―	2 (100.0)	2 (50.0)	6 (75.0)	―
4. More formula (baby food) than breastmilk	―	―	―	5 (100.0)	―	―	―	3 (100.0)

**Table 4:** Demographic and obstetric characteristics in interaction and video test (n = 18).

variable	Category	n (%)	Mean ± S.D.	Min.	Max.
Mother Age (year)			33.0 ± 4.8	26	41
Obstetric History	Primipara	8 (44.4)			
	Multipara	10 (55.6)			
Delivery	Vaginal delivery	18 (100.0)			
	Caesarian section	0 (0.0)			
Infant Age	6-7 months	18 (100.0)			
Infant Birth weight			3079.2 ± 363.3	2660	3920
Education	<4 years college guraduate	6 (33.3)			
	Guraduate 4 years college	11 (61.1)			
	Postgraduate	1 (5.6)			
Family Type	Nuclear family	14 (77.8)			
	Extended family	4 (22.2)			
Employment Situation	Working	3 (16.7)			
	Maternity leave	15 (83.3)			
	Housewife	0 (0.0)			
Menstruation	Postpartum amenorrhea	11 (61.1)			
	Restart	7 (38.9)			
EPDS (0-30)			4.4 ± 2.8	0	9
STAI (state-anxiety) (20-80)			37.9 ± 7.9	27	50
(trait-anxiety) (20-80)			34.4 ± 5.2	26	48
SRS-18 (0-54)			9.4 ± 5.2	0	21
MIBS-J (30-0)			1.4 ± 1.1	0	4

**Table 5:** Demographic and obstetric characteristics in interaction and video test classified by EPDS score.

Variable	Category	L–group (EPDS < 5) n = 9				H–group (EPDS ≥ 5) n = 9				P-value
		n (%)	Mean ± SD^†^	Min.	Max.	n (%)	Mean ± SD^†^	Min.	Max.	
Mother Age (year)			32.4 ± 4.7	26	39		33.7 ± 5.2	27	41	0.56
Obstetric History	Primipara	4 (44.4)				4 (44.4)				
	Multipara	5 (55.6)				5 (55.6)				
Delivery	Vaginal Delivery	9 (100.0)				9 (100.0)				
	Caesarian section	0 ( 0.0)				0 (0.0)				
Infant Birth weight			3190.2 ± 399.2	2690	3920		2968.2 ± 305.7	2660	3602	0.30
Education	4-year college graduate	4 (44.4)				2 (22.2)				
	Graduate 4 years college	5 (55.6)				6 (66.7)				
	Postgraduate	0 (0.0)				1 (11.1)				
Family Type	Nuclear family	8 (88.9)				6 (66.7)				
	Extended family	1 (11.1)				3 (33.3)				
Employment Situation	Working	2 (22.2)				1 (11.1)				
	Maternity leave	7 (77.8)				8 (88.9)				
	Housewife	0 ( 0.0)				0 ( 0.0)				
Menstruation	Postpartum amenorrhea	6 (66.7)				5 (55.6)				
	Restart	3 (33.3)				4 (44.4)				
EPDS (0-30)			2.1 ± 1.1	0	3		6.8 ± 1.6	5	9	<0.001^***^
STAI state (20-80)			31.9 ± 4.6	27	34		44.0 ± 5.5	34	50	<0.001^***^
STAI trait (20-80)			31.0 ± 2.6	26	34		37.9 ± 4.8	32	48	<0.001^***^
SRS-18 (0-54)			7.4 ± 4.7	0	15		11.4 ± 5.2	5	21	0.14
MIBS-J (30-0)			1.3 ± 1.4	0	4		1.6 ± 0.7	1	3	0.39

^†^SD, standard deviation, Significance level is shown p-value ^***^p < 0.001, (Mann-Whitney's U).

**Table 6:** Breastfeeding intensity in interaction and video test.

Breastfeeding methods	L–group (EPDS < 5) n = 9	H–group (EPDS ≥ 5) n = 9
	6-7 months	6-7 months
1. Exclusive breastmilk	―	―
2. More breastmilk than formula (baby food)	4 (44.4)	3 (33.3)
3. About the same amount of breastmilk and formula (baby food)	5 (55.6)	6 (66.7)
4. More formula (baby food) than breastmilk	―	―

### 2.5 Variables

The exposure variables were the three experimental tests: breastfeeding, mother–infant interaction, and infant video viewing. Psychological variables were assessed as described in Section 2.4. The primary outcome variable was the salivary OT concentration, analyzed to capture dynamic responses. Specifically, absolute values and relative change were calculated for all three tests, while the change in area under the curve (ΔAUC) was calculated only for the interaction and video tests. Correlational analyses between OT responses and psychological measures used relative change values at fixed time points (see section 2.12, 5 min for breastfeeding; 1 min for interaction/video) and ΔAUC (cumulative changes from time point 1 to time point 5).

EPDS scores were analyzed both as continuous variables and as categorical groupings (< 5 and ≥ 5) to compare OT reactivity between participants with lower and higher EPDS scores.

Potential confounding variables—including demographic and physiological/lifestyle factors detailed in Section 2.4 (e.g., maternal age, mode of delivery, and breastfeeding intensity)—were collected to describe participant characteristics. Given the exploratory nature of the study and the limited sample size, these variables were not included in multivariable analyses but are presented descriptively in **Tables 1**-**6**.

No diagnostic criteria were applicable, as all participants were healthy postpartum women without a diagnosis of PPD. No potential effect modifiers or interaction terms were prespecified in the analysis.

### 2.6 Bias

Potential sources of bias were considered. First, regarding selection bias, participants were mainly recruited during routine infant health checkups, either by being directly approached by pediatricians or by expressing interest themselves, with participation then confirmed by the pediatrician. Although this was not a random sampling, recruitment through pediatricians likely reduced self-selection bias. However, mothers who do not attend such visits may be underrepresented. Second, concerning sampling location, participants in the breastfeeding test could choose to collect samples either at home or at the university. To address potential differences, salivary OT responses were compared between these groups in this study, and no significant differences were observed (**Supplementary Figure 1**), indicating that sampling location was unlikely to have introduced systematic bias. Third, regarding sampling procedures, some participants provided both blood and saliva samples, while others provided saliva only. Since our previous findings showed that salivary OT levels increased even when blood sampling was conducted during all three tests (31), any potential influence of including participants with or without blood sampling in the present study is expected to be minimal, although a small effect cannot be ruled out. Fourth, although demographic and physiological/lifestyle variables were collected, they were not evaluated or included in the analyses of OT data. Last, as the psychological assessment data were self-reported, recall and social desirability bias cannot be ruled out.

### 2.7 Study size

A formal *a priori* power analysis was not conducted because the study was exploratory, aiming to identify potential patterns and associations rather than to detect specific effect sizes.

For the breastfeeding test, the final sample size was determined by feasibility. Recruitment was affected by coronavirus disease 2019 (COVID-19)–related restrictions in Japan, which limited the ability to recruit mothers at comparable postpartum stages. A total of 69 mothers at 1, 3, 6–7, and 12 months postpartum participated in the breastfeeding experiment, and 61 were included in the final analysis of OT and psychological test results *(*1 month, n *=* 12; 3 months, n = 17; 6–7 months, n *=* 24; and 12 months, n = 8). Among these, 38 provided saliva samples, and 23 provided both saliva and plasma samples. The exclusion criteria for saliva data analysis are shown in **Figure 1A**.

For the interaction and video tests, we built on our previous study (31), in which both tests elicited clear increases in salivary OT levels among postpartum mothers with low EPDS scores (< 3). The present study aimed to examine whether similar OT responses would be observed in a broader group of mothers. For these tests, the sample size was approximately doubled relative to the previous study. A total of 21 mothers at 6–7 months postpartum participated, and 18 were included in the final analysis of OT and psychological test results. Of these, 7 provided saliva only, and 11 provided both saliva and plasma samples. The exclusion criteria for these tests are shown in **Figure 1B**.

### 2.8 Sampling procedure

#### 2.8.1 Breastfeeding test

Experiments were conducted either at the university or at participants’ homes. At the university, mothers arrived one hour before the experiment. An indwelling needle was inserted into a forearm vein for serial blood sampling, after which participants completed questionnaires and waited for the experiment to begin. We ensured that at least two hours had passed since the previous feeding. Sampling points are shown in **Figure 1C**. After breastfeeding, mothers spent time with their infants as usual until the subsequent sampling points. Mothers who opted for saliva-only collection followed written instructions and used standardized kits at home. All sampling procedures at home were supervised by trained researchers via written instructions and follow-up communication to ensure procedural consistency.

#### 2.8.2 Interaction and video tests

Mothers at 6–7 months postpartum participated in both tests. A researcher with phlebotomy qualifications visited their homes one hour before the experiments to provide explanations and obtain informed consent. Participants chose whether to provide saliva samples alone or both saliva and blood samples. For those who agreed to provide blood samples, an indwelling needle was inserted into a forearm vein. Sampling points are shown in **Figure 1D**. The interaction test was conducted first, followed by the video test at least one week later, in accordance with previously established protocols (31).

### 2.9 Saliva and blood collection

Sample collection was conducted only when participants were in good health, without infectious diseases, ongoing dental treatments, or oral conditions, to ensure that samples were obtained under healthy conditions. All samples were collected between 9:00 a.m. and 11:00 a.m. Participants were instructed to refrain from eating or drinking (except water) for at least 1.5 hours before baseline sampling and to avoid stress, vigorous exercise, or heavy physical activity during this period. Handling and storage of saliva samples followed previously described procedures. Home-collected saliva samples were stored in participants’ home freezers and later returned to the researchers via temperature-controlled delivery.

### 2.10 OT quantification

Salivary OT concentrations were measured using an OT-ELISA kit (Cat. No. ADI-901-153A-0001; Enzo Life Sciences, Farmingdale, NY, USA) according to the manufacturer’s protocol and previous reports (31–33). The detection sensitivity was 15 pg/mL, and the measurement range was 15.6–1000 pg/mL. Samples were measured in duplicate, and optical density was read at 405 nm using a microplate reader (Bio-Rad, Richmond, CA, USA). Concentrations were calculated from standard curves. Intra- and inter-assay coefficients were < 4.2% and < 6.9%, respectively. Plasma OT was not analyzed in this study due to the small number of blood donors; thus, this study focused on salivary OT, which was consistently collected across participants.

### 2.11 Data analysis

All demographic and physiological/lifestyle variables were complete, with no missing data. A small number of participants had missing psychometric data, and the corresponding saliva data were excluded from analyses requiring correlation with psychological measures. Data were also excluded according to predefined criteria, including breastfeeding durations shorter than 5 min or longer than 30 min, failure to collect saliva samples at all sampling points, insufficient saliva volume for OT assay, or absolute values statistically identified as outliers.

The number of excluded cases for each criterion is summarized in the exclusion flowchart (**Figure 1**). Relative change values were calculated using the −10 min measurement as the basal value, normalized to 1.0 (i.e., basal/basal), and all subsequent time points were expressed as ratios relative to the basal value (each time point/basal). To assess the net changes in salivary OT levels, the change in area under the curve (ΔAUC) was calculated using the trapezoidal method. The −10 min time point was defined as the basal. For each participant, basal values were subtracted from all subsequent time points, and ΔAUC was calculated from these basal-adjusted values over time points 1 to 5. The resulting ΔAUC represents the cumulative change in OT concentration relative to baseline.

To further explore associations between OT reactivity and psychological measures, EPDS and STAI score distributions were compared between mothers showing no increase in salivary OT (relative change ≤ 1) and those showing an increase (relative change > 1) at the 5-min time point. Interquartile ranges (IQRs) were computed for each group to summarize score dispersion within each group.

Correlational analyses were conducted using the relative change values of OT—analyzed at the 5-min time point in the breastfeeding test and the 1-min time point in the interaction and video tests—in relation to psychological measures, including EPDS, STAI, SRS-18, and MIBS-J. In addition, associations between the change in area under the curve (ΔAUC) and psychological measures were examined in the interaction and video tests.

For each test, analyses were first performed on the entire sample. Subsequently, participants were divided into two groups based on EPDS scores (EPDS < 5 and EPDS ≥ 5). Comparative analyses were then conducted between these two groups to examine differences in the absolute value of OT and relative changes.

### 2.12 Statistical analysis

All statistical analyses were performed using Prism 9.5.1 (GraphPad Software Inc., San Diego, CA, USA). As absolute OT concentrations did not follow a normal distribution, a log_10_ transformation was applied before analysis. Statistical analyses were conducted using two-way repeated-measures ANOVA. For *post hoc* analysis, Tukey’s multiple comparison test was used for within-group comparisons, and Sidak’s *post hoc* test for between-group comparisons at specific time points. Since RC values were not normally distributed, they were analyzed using the Friedman test followed by Dunn’s *post hoc* test. Pearson’s or Spearman’s correlation coefficients were assessed for relative change values (breastfeeding, 5 min; interaction/video, 1 min) and psychometric scores. Outliers were identified using the robust regression and outlier removal (ROUT) method (Q = 1%). The changes in absolute and relative OT concentrations over time were compared between two EPDS groups: EPDS < 5 and EPDS ≥ 5, using two-way repeated-measures ANOVA followed by the same *post hoc* procedures described above. Statistical significance was set at *p* < 0.05. Potential confounding factors were collected; however, given the exploratory nature and limited sample size, multivariable adjustment was not performed. Statistical significance was set at *p* < 0.05, and all data were mean ± SEM.

## 3 Results

### 3.1 Changes in salivary OT concentrations during breastfeeding test

A total of 69 mothers participated in the breastfeeding test, and data from 61 mothers were included in the final analysis. Demographic characteristics of the mothers are shown in **Table 1**. The mean maternal age was 33.1 ± 4.2 years, and 24.6% were primiparous. The postpartum period at testing ranged from 1 to 12 months, and the average infant birth weight was 3014.3 ± 353.8 g. Most mothers lived in a nuclear family structure (86.9%), had completed a four-year college degree or higher (62.3%), and were on maternity leave or homemakers (91.8%) at the time of testing. Twenty-three percent had resumed menstruation, while 77.0% remained amenorrheic.

**Figure 2:**
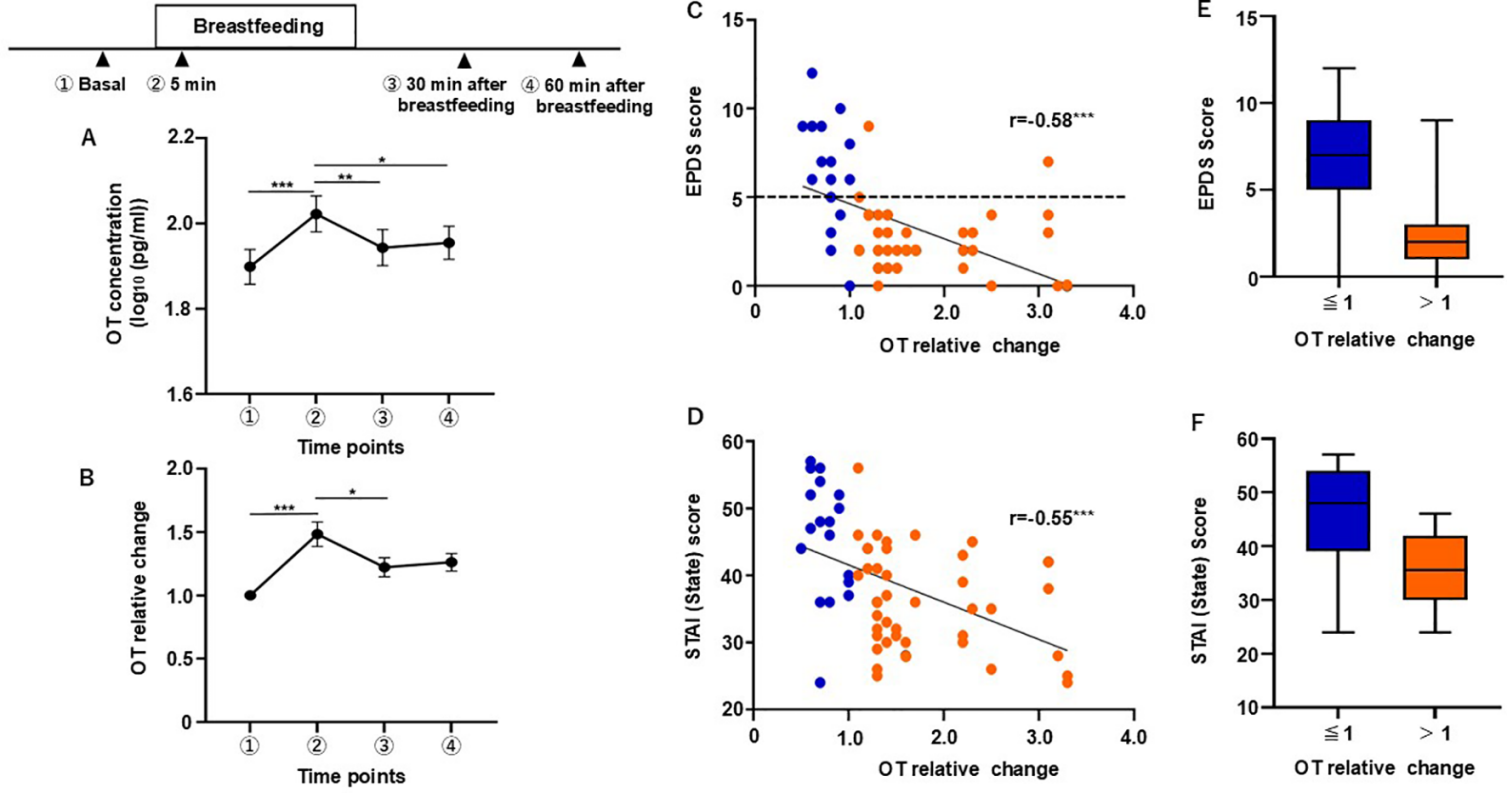
Correlation between scores of psychological scale scores and the relative change (RC) in OT concentration observed at the time point of 5 min after breastfeeding. **(A)** Changes in salivary OT concentration during the breastfeeding test. **(B)** RC in salivary OT. **(C)** Correlation between the RC in salivary OT levels at 5 minutes and EPDS scores. **(D)** Correlation between the RC in salivary OT levels at 5 minutes and STAI (State Anxiety) score. **(E)** Subjects were divided into an increase group (RC > 1) and a non-increase group (RC ≤ 1) based on the RC of salivary oxytocin during breastfeeding at the 5-minute time point. The interquartile range (IQR) of EPDS scores for each group is shown. **(F)** The same analysis was conducted for STAI (State Anxiety) scores. n = 61. Mean ± SEM. *p < 0.05, **p < 0.01, ***p < 0.001.

The salivary OT level increased during breastfeeding but then decreased nearly back to the basal level 30 min after breastfeeding ended (**Figure 2A**). This fluctuation over the course of breastfeeding was found to be significant using a one-way repeated measures ANOVA test (F[2.804, 168.2] = 8.630, *p* < 0.001). Tukey’s multiple comparison test revealed a significant difference in salivary OT concentration at 5 min after breastfeeding compared with the basal level, and the levels 30 and 60 min after breastfeeding (basal vs. 5 min, *p* < 0.001; 5 min vs. 30 min, *p* = 0.009; 5 min vs. 60 min, *p* = 0.043).

Then we analyzed the relative change in salivary OT concentration. There was a significant difference between time points during breastfeeding (Friedman’s test, χ² [3] = 19.93, *p* < 0.001), (**Figure 2B**). Dunn’s *post hoc* analysis showed that the salivary OT level at 5 min was significantly different from the basal level and that at 30 min after breastfeeding (basal vs. 5 min, *p* < 0.001; 5 min vs. 30 min, *p* = 0.01).

### 3.2 Association between relative changes in salivary OT during breastfeeding and psychological scale scores

We then focused on the correlation between EPDS scores and the relative change (RC) in OT concentration at 5 min after breastfeeding, using the same dataset described in Section 3.1. A statistically significant negative correlation was found between the EPDS scores and the RC value at 5 min time point, as determined by Spearman’s rank correlation test (ρ[61] = −0.583, *p* < 0.001, n = 61), (**Figure 2C**). Furthermore, a significant negative correlation was found in the subgroups of postpartum mothers (**Supplementary Figure 2**), although the group of mothers at 1 month postpartum showed only a tendency for such a correlation (**Supplementary Figure 2A**). We also examined the association between other psychological scale scores and RC value (**Table 7**). A significant negative correlation was found between RC value and STAI (state anxiety, ρ[61)]= −0.548, *p* < 0.001), (**Figure 2D** and **Table 7**).

**Table 7:** Correlation between the relative change in salivary OT during each test and psychological scale scores.

Breastfeeding methods	Psychological scale scores				
	EPDS	STAI state	STAI trait	SRS-18	MIBS^†^
During breastfeeding^†^	-0.58***	-0.55^***^	-0.07	-0.23	0.01
During interaction test	-0.83***	-0.80^**^	-0.53^*^	-0.61	-0.15
During video test	-0.55**	-0.43	-0.41	-0.30	-0.11

Pearson’s correlation coefficients, unless noted. *p < 0.05, **p < 0.01, ***p < 0.001

†Spearman’s rank correlation test. ^***^p < 0.001

We further noted that 80% of postpartum mothers who did not show an increase in salivary OT concentration (RC value of ≤ 1) during breastfeeding had an EPDS score of ≥ 5 (**Figure 2C**, blue plots). Therefore, we examined the interquartile range (IQR) of the EPDS scores for mothers with an RC value of ≤ 1 and for those with > 1. The IQR of the EPDS scores in those with an RC value of ≤ 1 was 5–9, whereas for those with an RC value of > 1 was 1–3, being notably different (**Figure 2E**). Conversely, the IQR for STAI scores for mothers with an RC value of ≤ 1 was 39–54, whereas for those with an RC value of > 1 it was 30–42. Unlike for the EPDS scores, the IQR ranges for the STAI (State) scores of the two groups overlapped (**Figure 2F**).

**Figure 3:**
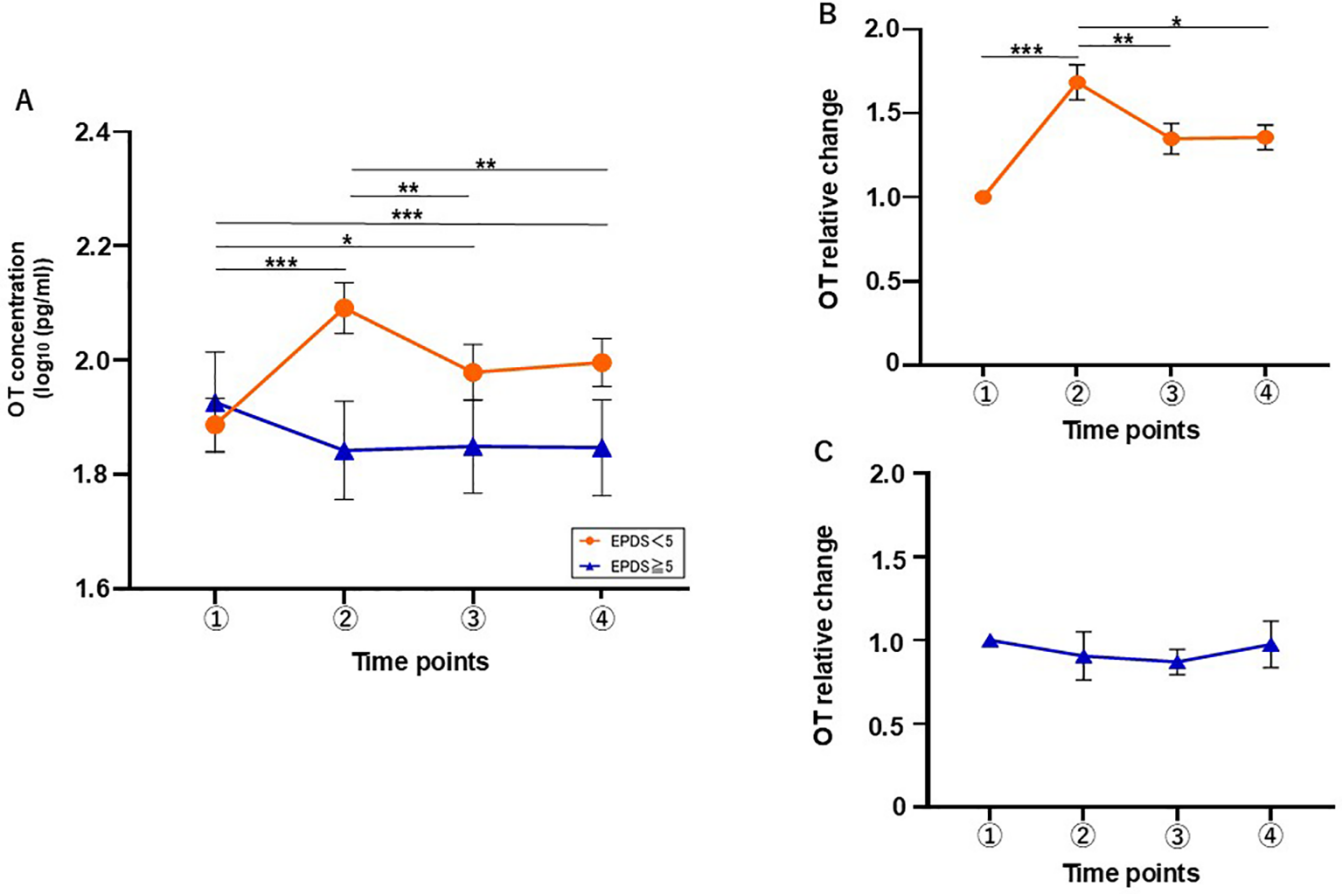
Changes in salivary OT concentration during the breastfeeding test classified by EPDS score. **(A)** The concentration of salivary OT in mothers with EPDS scores of <5 (L-group) and ≥ 5 (H-group). n = 61. **(B)** Relative change of salivary OT in the L-group (n = 44). **(C)** Relative change of salivary OT in the H-group (n = 17). Mean ± SEM. *p < 0.05, **p < 0.01, ***p < 0.001.

Given these findings, we set the cutoff score for EPDS at 5 and divided the postpartum mothers into two groups: ≥ 5 (high-score group, H-group; n = 17) and < 5 (low-score group, L-group; n = 44). The same dataset used to present the demographic characteristics of mothers in **Table 1** was reanalyzed, with participants categorized into high (EPDS ≥ 5) and low (EPDS < 5) groups (**Table 2**). We then reanalyzed their OT levels during breastfeeding. The L-group showed an increase at the time point of 5 min after breastfeeding, whereas no increase was observed in the H-group (**Figure 3A**). Two-way repeated measures ANOVA was conducted to examine the effects of time and group on OT response during breastfeeding in the L-group. There were no significant main effects for either time (F[2.798, 165.1] = 2.245, η^2^p = 0.037, *p* = 0.089) or group (F[1, 59] = 2.090, η^2^p = 0.207, *p* = 0.154). However, there was a significant interaction between time and group (F[3, 177] = 11.12, η^2^p = 0.159, *p* < 0.001). In the L-group, *post hoc* Tukey’s multiple comparison test revealed that there were significant differences between the basal level and those at all other time points (vs. 5 min, *p* < 0.001, vs. 30 min, *p* = 0.013, vs. 60 min, *p* < 0.001) and between 5 min after breastfeeding and subsequent time points (vs. 30 min, p = 0.002, vs. 60 min, *p* = 0.008). In the H-group, no significant differences between time points were found. Together, these results suggest that an OT increase during breastfeeding occurs predominantly among mothers in the L-group.

The RC of OT level showed an increase at 5 min after breastfeeding. Friedman’s test revealed significant differences across time points in the L-group (χ²[3] = 28.0, *p* < 0.001), (**Figure 3B**). Dunn’s *post hoc* test showed a significant increase in the RC value of salivary OT at the time point of 5 min after breastfeeding compared with the basal level (basal vs. 5 min, p < 0.001) and a significant decrease between 5 min and 30 and 60 min after breastfeeding (vs. 30 min, *p* < 0.002, vs. 60 min, *p* = 0.046). In contrast, the H-group showed no significant RC (**Figure 3C**).

The breastfeeding test was conducted either at home or in a university room setting. We examined whether the sample collection location (home versus university room), which was consistent across L- and H-groups, influenced the OT response. In the L-group, there was a clear increase in OT levels at 5 min after breastfeeding in both settings (n = 32 and 12 for the home and university room settings, respectively), (**Supplementary Figure 1A**). In contrast, no such increase was observed in either setting within the H-group (n = 10 and 7 for the home and university room settings, respectively), (**Supplementary Figure 1B**). Two-way repeated-measures ANOVA was conducted separately for the L- and H-groups to examine the effects of time and collection setting on OT levels. In the L-group, a significant main effect of time was observed (F[2.664, 111.9] = 17.69, η^2^p = 0.296, *p* < 0.001), with no significant effects of the collection setting or time × setting interaction (F[3, 126] = 0.498, η^2^p = 0.106, *p* = 0.684). *Post hoc* analysis confirmed a significant increase in OT levels at 5 min after breastfeeding, which was consistent across both collection settings (**Supplementary Figure 2A**). In the H-group, there were no significant main effects of time (F[2.199, 32.98] = 1.341, η^2^p = 0.082, *p* = 0.277), collection setting (F[1, 15] = 0.985, η^2^p = 0.342, *p* = 0.337), or their interaction (F[3, 45] = 0.218, η^2^p = 0.014, *p* = 0.884), (**Supplementary Figure 2B**). In the L- and H-groups, OT levels did not differ between the collection settings at any time point. These findings suggest that the sample collection setting did not significantly influence OT responses in either group.

### 3.3 Association between the relative changes in salivary OT during the interaction and video tests and EPDS scores

We evaluated the OT response of mothers at 6–7 months postpartum during direct (interaction test) and indirect (video test) encounters with an infant as previously reported (31). The demographic characteristics of these mothers are shown in **Table 4**. Across all participants, no significant changes in salivary OT concentrations were observed over time during either the interaction or video test, as determined by one-way repeated-measures ANOVA (interaction test: F[3.640, 61.88] = 1.703, *p* = 0.166; video test: F[5, 102] = 0.150, *p* = 0.100, n = 18 and 18 for the interaction and video test, respectively), (**Supplementary Figures 3A, B**). However, during the breastfeeding test, participants’ OT responses differed, with some exhibiting a marked increase and others showing little or no change according to the EPDS score.

Given this, to further examine whether OT response during the interaction and video tests was associated with psychological scale scores, we tested correlations between psychological measures (e.g., EPDS and STAI) and two indices of OT response: ΔAUC (net change from the basal level) and the RC (1 min/basal). We reanalyzed the dataset (the same dataset used for the demographic characteristics in **Table 4**), categorizing participants into high (EPDS ≥ 5) and low (EPDS < 5) groups (**Table 5**). No significant associations were observed between EPDS scores and ΔAUC (**Supplementary Figures 4** and **5**). However, a significant negative correlation was found between EPDS scores and the 1-min RC value, of both interaction and video tests indicating that higher EPDS scores were associated with reduced OT response (**Supplementary Figure 6**). These findings suggest that brief, time-specific responses such as RC may better capture individual differences related to psychological state than summary indices such as ΔAUC. Pearson’s or Spearman’s correlation coefficients between these OT measures and other psychological scales are summarized in **Supplementary Figure 6** and **Supplementary Table 1**.

**Figure 4:**
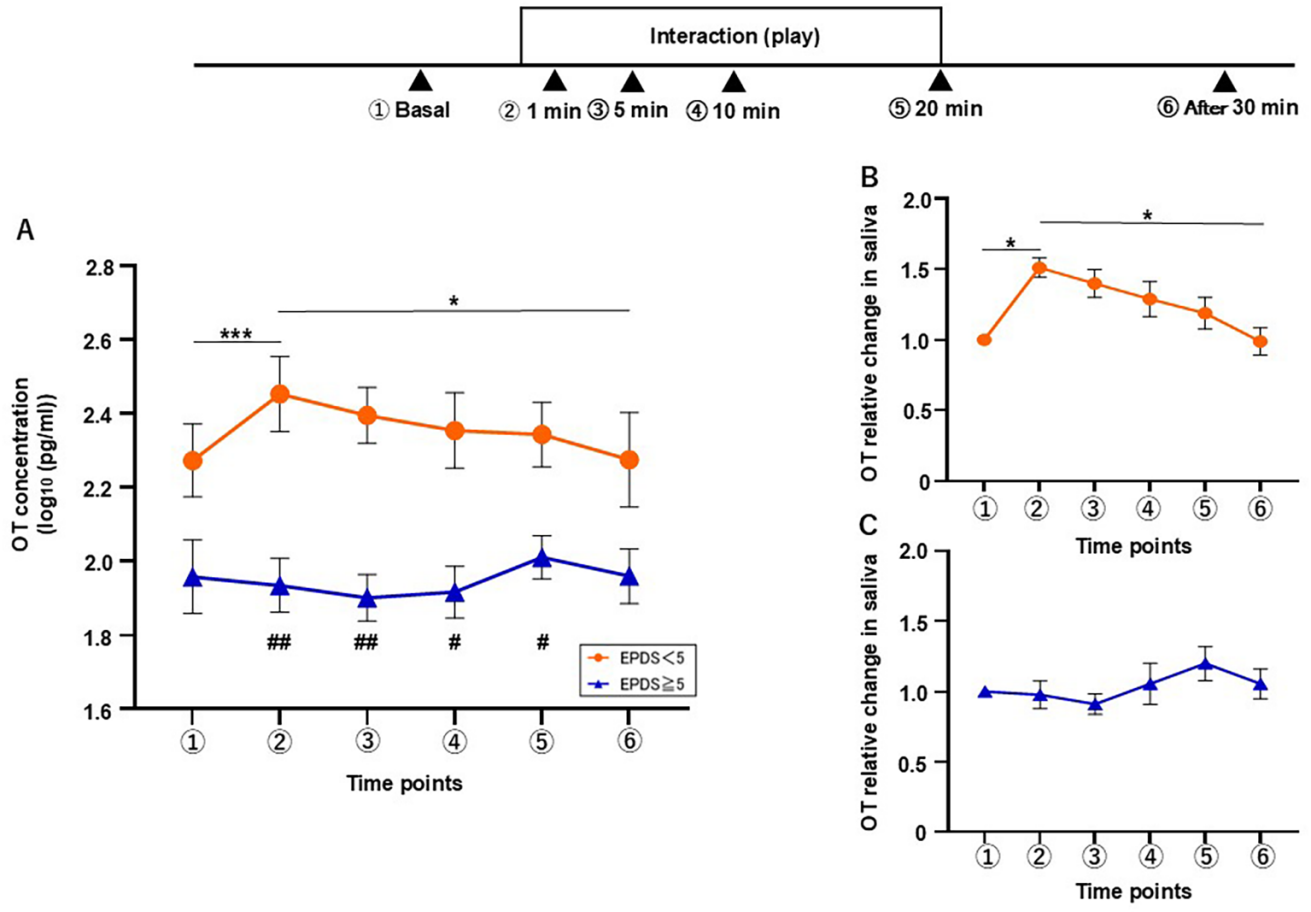
Changes in salivary OT concentration during the interaction test were classified by EPDS score. **(A)** The concentration of salivary OT in mothers with EPDS scores of < 5 (L-group) and ≥ 5 (H-group) (n = 18). **(B)** Relative change of salivary OT in the L-group (n = 9). **(C)** Relative change of salivary OT in the H-group (n = 9). Mean ± SEM. Within-group timepoints, *p < 0.05, ***p < 0.001. Between-group timepoints, #p < 0.05, ##p < 0.01.

We then wondered whether these responses would differ between the mothers who scored < 5 (L-group; n = 9) and those who scored ≥ 5 (H-group; n = 9) as we observed in the breastfeeding test. In the interaction test, the L-group showed an increase in the OT level as early as 1 min after the interaction, whereas no increase was observed in the H-group. Two-way repeated measures ANOVA was conducted to examine the effects of time and group on OT response during the interaction test (**Figure 4A**). This revealed significant main effects for group (F[1, 16] = 11.08, η^2^p = 0.882, *p* = 0.004) and time (F[3.657, 58.51] = 2.740, η^2^p = 0.115, *p* = 0.041), and a significant interaction of time × group (F[5, 80] = 3.289, η^2^p = 0.231, *p* = 0.009). Additionally, *post hoc* Tukey’s multiple comparison test revealed significant differences between the basal level and that at 1 min (vs. 1 min, *p* < 0.001) and between the time points of 1 min and 30 min (*p* = 0.043) in the L-group. No significant differences were found among any of the time points in the H-group. Furthermore, Sidak’s *post hoc* test showed a significant difference in the OT level between L- and H-groups at each time point from 1 to 20 min (1 min, *p* = 0.009, 5 min, *p* = 0.003, 10 min, *p* = 0.019, 20 min, *p* = 0.046).

The relative change in the salivary OT level showed a significant increase during the interaction with the infant in the L-group (Friedman’s test, χ²[5] = 18.73, *p* = 0.022; **Figure 4B**), whereas no significance was detected in the H-group (χ²[5] = 10.06, *p* = 0.060; **Figure 4C**). Dunn’s *post hoc* test revealed significant differences in this variable between the basal level and that at 1 min (p = 0.020), and between the levels at 1 min and 30 min (vs. 30 min, *p* = 0.025) in the L-group. No significant differences were found among any of the time points in the H-group.

**Figure 5:**
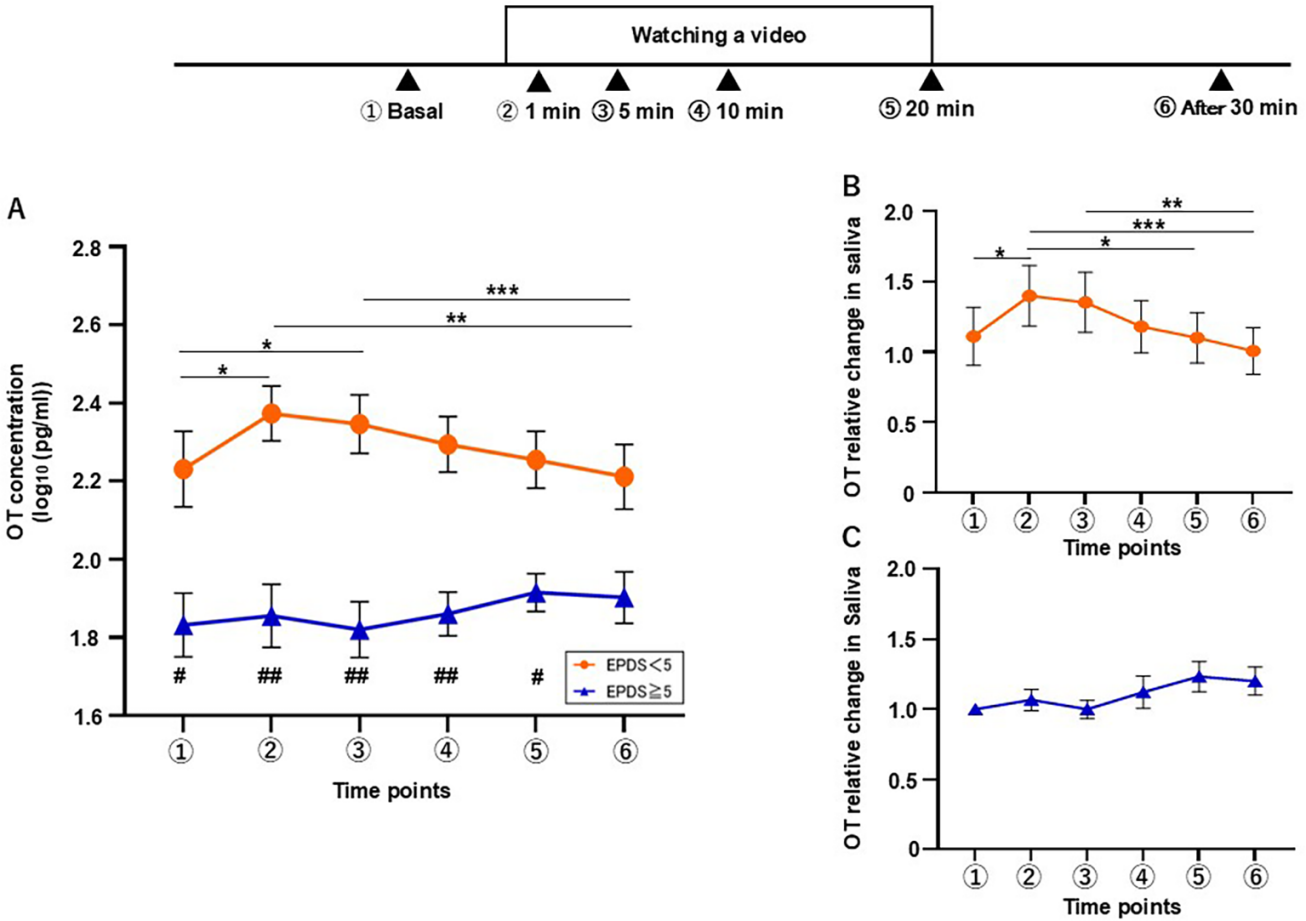
Changes in salivary OT concentration during the video test were classified by EPDS score. **(A)** The concentration of salivary OT in mothers with EPDS scores of < 5 (L-group) and ≥ 5 (H-group) (n = 18). **(B)** Relative change of salivary OT in the L-group (n = 9). **(C)** Relative change of salivary OT in the H-group (n = 9). Mean ± SEM. Within-group time points, *p < 0.05, **p < 0.01, ***p < 0.001. Between-group timepoints, #p < 0.05, ##p < 0.01.

In the video test, as previously reported (31), the L-group showed an increase in OT level at 1 min, which then gradually decreased toward the end of the test (n = 9, **Figure 5A**). Two-way repeated measures ANOVA revealed significant main effects for group (F[1, 16] = 17.64, η^2^p = 0.929, *p* = 0.001) and time (F[2.958, 47.32] = 3.087, η^2^p = 0.162, *p* = 0.037), and a significant interaction of time × group (F[5, 80] = 7.88, η^2^p = 0.330, *p* < 0.001). Significant differences were found by *post hoc* Tukey’s multiple comparison test in the L-group for the time points of 1 and 5 min when compared with the basal level (vs. 1 min, *p* = 0.013, 5 min, *p* = 0.015) and for 1 and 5 min when compared with the level at 30 min after the test (vs. 1 min, *p* = 0.001, vs. 5 min, *p* < 0.001) in the L-group. No significant differences were found among any of the time points in the H-group (n = 9). Furthermore, Sidak’s *post hoc* test revealed a significantly lower level of OT in the H-group than in the L-group at all time points, except for the last timepoint (basal, *p* = 0.038, 1 min, *p* = 0.001, 5 min, *p* = 0.001, 10 min, *p* = 0.001, 20 min, *p* = 0.01).

The RC in salivary OT concentration during the video test showed a clear increase at the time point of 1 min in the L-group, whereas the H-group exhibited a weak response. According to Friedman’s test, the L-group showed a significant change in this variable (χ²[5] = 28.90, *p* < 0.001; **Figure 5B**), while the H-group also showed a significant change, albeit a less significant one (χ²[5] = 11.47, *p* < 0.043; **Figure 5C**). In the L-group, Dunn’s *post hoc* test revealed significant differences in this variable between the basal level and that at 1 min (*p* = 0.038), and between 1 min and 20 and 30 min after the video test (vs. 20 min, *p* < 0.038; vs. 30 min after video test, *p* = 0.001). There was also a significant difference between 5 and 30 minutes after the video test in the L-group (*p* = 0.002). Meanwhile, no significant differences were found between any timepoints in the H-group.

**Figure 6:**
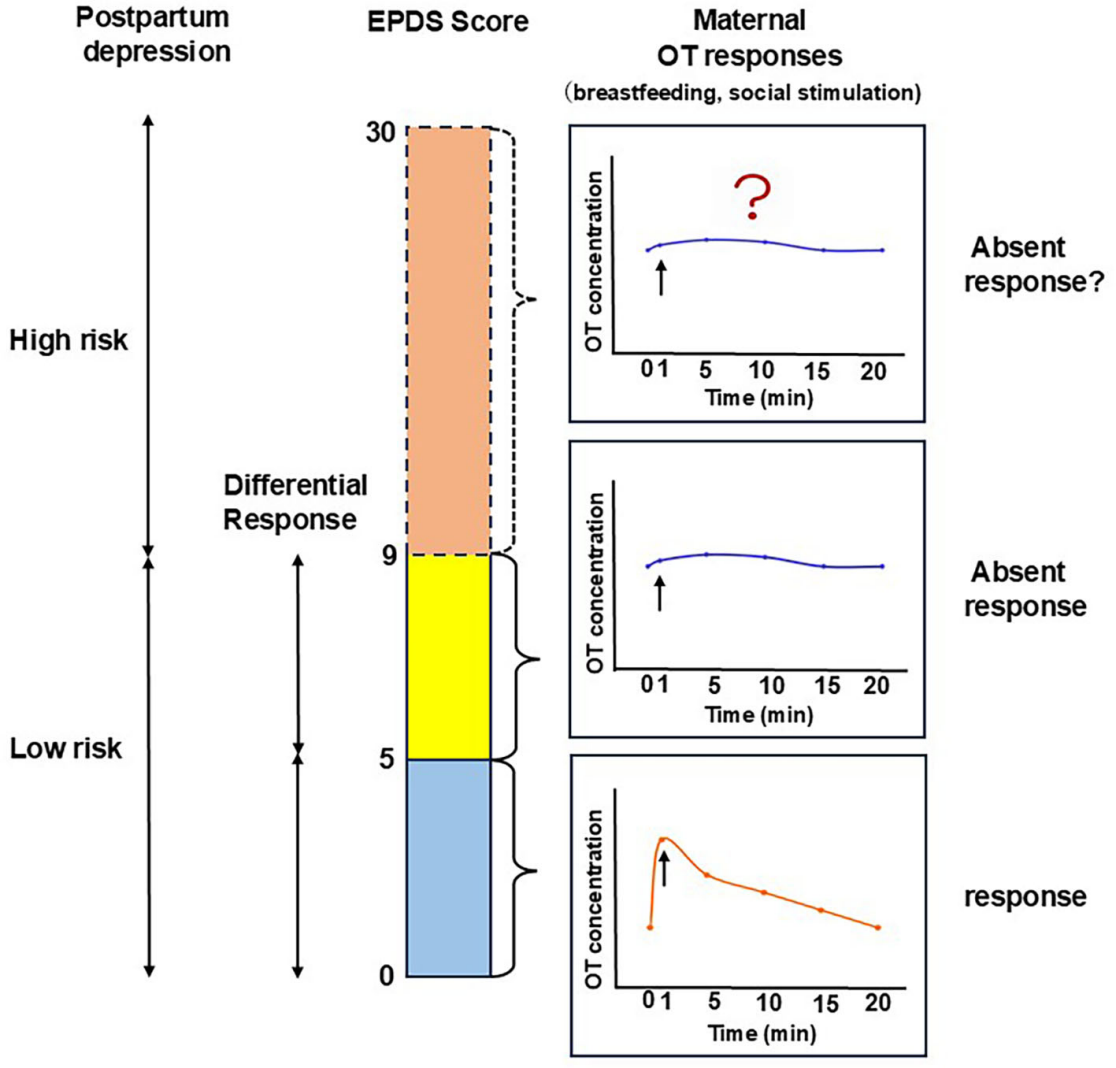
A schematic illustration of the main findings showing the association between salivary OT responses to infant stimuli and EPDS scores among mothers without a diagnosis of postpartum depression.

In summary, this study explored the relationship between EPDS scores and salivary OT responses to infants among postpartum Japanese mothers without clinical PPD diagnoses. The results showed that maternal OT responses to both physiological and social infant stimuli differed according to psychological state, with a possible transition in response patterns observed around an EPDS score of 5, below the screening cutoff (**Figure 6**).

## 4 Discussion

This study aimed to determine whether postpartum mothers without a clinical diagnosis of PPD vary in OT responses to infant-related stimuli according to maternal psychological state, as assessed by EPDS scores. We found that maternal OT responses changed with psychological state, showing a transition in response patterns around an EPDS score of 5, which is below the typical screening cutoff (**Figure 6**). Specifically, mothers with an EPDS score of < 5 (L-group) exhibited a rapid increase in OT when breastfeeding or upon encountering their infant. In contrast, mothers with higher EPDS scores (≥ 5, H-group) hardly showed any increase in OT responses. These results raise the possibility that alterations in OT responsiveness may emerge even among mothers not reaching the screening threshold, pointing to subtle biological changes that could precede clinically recognizable depressive symptoms.

Previous studies reported an inverse relationship between basal plasma OT levels in late pregnancy and/or the postpartum period and an increase in postpartum depressive symptoms (21). Additionally, an inverse correlation between OT level during breastfeeding and depressive states, as measured by the EPDS, and a similar association with state anxiety levels (STAI-state) has been reported (20, 34). However, to the best of our knowledge, no studies have shown the association between the social cue-elicited maternal OT response and EPDS scores or documented differences in variation in maternal OT responses within the low-risk range of EPDS scores. Therefore, this study is the first to suggest that, even when EPDS scores fall below the screening cutoff, physiological variation exists, and differences in maternal OT responsiveness to social and physiological stimuli may reflect subtle psychological differences within the low-risk range. Although causality cannot be inferred, the observed differences in OT response patterns may reflect early biological changes linked to postpartum mental health.

The H-group mothers showed lower basal OT levels than the L-group mothers, but significant group differences appeared only in the video test and not in the interaction test. Since the same participants took part in both tests, this inconsistency suggests that basal OT levels may vary depending on daily or situational factors, rather than representing a consistent difference between groups. This variability suggests that obtaining a reliable estimate of an individual’s basal OT level is challenging, and that changes in OT in response to specific stimuli may better reflect mothers’ emotional states and psychological well-being.

The OT level acutely (within 1 min) increased after interaction with their infant or watching a video of them. It has been shown that interactions within bonded relationships, such as between mother/father and infant, lead to an increase in OT levels. For instance, Feldman et al. showed that OT levels increased in both mother’s and father’s saliva OT levels after free play with their 4–6-month-old infants (26). However, to the best of our knowledge, no studies have investigated the time course of OT release in more detail. Our current study, together with our prior research (31), demonstrated that maternal OT levels can increase very rapidly within 1 min of direct interaction with their infant or watching a video of them, which returned to baseline levels after 20 min. Meanwhile, McNeilly et al. reported not only that the mother’s OT level increases during suckling, but that a baby’s cry also elevates the OT level (35). Therefore, a child’s social salience may be particularly prominent in the mother’s perception during the postpartum period. This rapid rise and subsequent decrease in OT levels revealed in our study may provide insight into the dynamic nature of the OTergic system, suggesting that it may enable mothers to adapt to rapid changes in their infants. Nevertheless, it remains unclear whether such dynamics are specific to the early postpartum period. Further studies examining the response at different stages of motherhood would be valuable to understand how the dynamic OT response develops over time.

OT responses to infant-related stimuli in the postpartum period may be influenced by both affective mother–infant bonding processes and physiological adaptations associated with breastfeeding. Breastfeeding involves multiple neuroendocrine events, including pulsatile OT release to support the milk-ejection reflex (36), elevated prolactin activity (37, 38), and attenuated HPA-axis reactivity (22, 39). Repeated OT release during feeding may contribute to transient reductions in OT responsivity (e.g., release fatigue), consistent with evidence from prior studies showing OT/OTR desensitization following elevated or prolonged OT exposure (40). Similarly, exposure to synthetic OT during labor has been associated with alterations in OT levels during breastfeeding (41, 42). Therefore, such variation in lactation patterns and physiological states, including hormonal status, may influence baseline OT levels and responsivity to infant cues. In our present study, all participants were breastfeeding and the extent and nature of breastfeeding varied across individuals. Information on breastfeeding status (e.g., predominantly breastfeeding, mixed feeding, or primarily formula/food feeding with some breastfeeding), postpartum stage, and hormonal cyclicity (i.e., whether menstruation had resumed) was collected as part of the demographic data, but was not included in the statistical analyses. Future research should incorporate these variables to better understand their associations with OT dynamics in the postpartum period.

In this study, we measured salivary OT concentrations using a commercially available ELISA kit (Enzo Life Sciences) without an extraction step. Consequently, the detected concentrations were higher than those typically reported in studies that applied extraction procedures. This discrepancy likely reflects methodological differences: the extraction process can substantially reduce the measured concentrations by removing the protein-bound OT and other molecular forms, whereas the unprocessed samples may include the free and bound forms, as well as the precursor molecules or OT complexed with the carrier proteins (43, 44). There is currently no established consensus on whether measuring free or bound OT more accurately reflects biologically active levels. Both approaches may carry distinct advantages and limitations, and their suitability may vary depending on the study design and practical considerations. In our case, although the absolute concentrations may not correspond to those obtained through the extracted methods, we applied the same nonextraction protocol consistently across all participants and time points. This allowed us to detect physiologically meaningful patterns of change—such as increases during breastfeeding—that were consistent with established findings (31). Therefore, while caution is warranted in interpreting absolute levels, the relative within-subject changes captured by our protocol are likely to be a reliable reflection of OT dynamics.

Our study had several limitations. First, the number of subjects who participated in the interaction and video tests was small. In addition, the mothers participating in the breastfeeding tests varied significantly in terms of their stage in the postpartum period, ranging from 1 to 12 months. This heterogeneity may introduce limited statistical power and the precision of the estimates. Future research in which the covariates are controlled as much as possible should be planned to ensure more accurate results. Second, the participants in the interaction and video tests did not include mothers who interrupted breastfeeding. Consequently, it is not clear whether similar OT responses would be observed in mothers who are not breastfeeding. There is thus a need for further validation with non-breastfeeding mothers. Third, this study was conducted with Japanese mothers, and cultural factors may influence both emotional experiences and hormonal responses, so examining these associations in other cultural settings will be important. Fourth, because all stimuli in this study involved participants’ own infants, the present design cannot determine whether the diminished OT response in the H-group reflects a general alteration related to affective dysregulation or a specific reduction in maternal–infant social responsiveness. Future studies, including responses to unfamiliar infants and non-social or neutral stimuli, will be necessary to clarify this distinction. Last, this study was not preregistered, and the key finding—namely, the absence of OT reactivity in some participants—was not anticipated. Accordingly, the analyses were exploratory in nature, and the present results should be interpreted cautiously given the modest sample size. Future studies aiming to replicate or formally test these individual differences should adopt preregistered designs to enhance transparency and reproducibility.

In conclusion, our findings showed that salivary oxytocin (OT) responses to infant stimuli vary according to EPDS scores among postpartum Japanese mothers without clinically diagnosed PPD. These differences, observed below the EPDS screening cutoff, may reflect early biological sensitivity related to vulnerability to PPD. Although the mechanisms determining who eventually develops PPD remain unclear, our results suggest that combining assessments of OT reactivity with EPDS screening may enhance the early identification of at-risk individuals. Further, these findings may highlight the potential role of OT dynamics as a biological marker of subtle emotional changes during the early postpartum period.

## Data Availability

The raw data supporting the conclusions of this article will be made available by the authors, without undue reservation.
