# Correction: Salivary oxytocin responses to infant stimuli vary by EPDS scores among postpartum Japanese mothers without clinically diagnosed postpartum depression

**DOI:** 10.3389/fendo.2025.1770642

**Published:** 2026-01-13

**Authors:** Kana Minami, Haruhiro Higashida, Shigeru Yokoyama, Takahiro Tsuji, Naomi Kagami, Chiharu Tsuji

**Affiliations:** 1 Research Center for Child Mental Development, Kanazawa University, Kanazawa, Japan; 2 Department of Health Development Nursing, Institute of Medical, Pharmaceutical and Health Sciences, Kanazawa University, Kanazawa, Japan; 3 Department of Socioneurosciences, United Graduate School of Child Development, Osaka University, Kanazawa University, Hamamatsu University School of Medicine, Chiba University, and University of Fukui, Kanazawa, Japan; 4 Department of Ophthalmology, Faculty of Medical Sciences, University of Fukui, Fukui, Japan

**Keywords:** postpartum depression, oxytocin, EPDS, mother, saliva

In the published article, there was a mistake in the affiliations 3-7. These five affiliations appeared as follows:

^3^Department of Socioneurosciences, United Graduate School of Child Development, Osaka University, Kanazawa, Japan

^4^Kanazawa University, Kanazawa, Japan

^5^Hamamatsu University School of Medicine, Kanazawa, Japan

^6^Chiba University, Kanazawa, Japan

^7^University of Fukui, Kanazawa, Japan

These affiliations have now been consolidated into one affiliation:

^3^Department of Socioneurosciences, United Graduate School of Child Development, Osaka University, Kanazawa University, Hamamatsu University School of Medicine, Chiba University, and University of Fukui, Kanazawa, Japan

The previous affiliation 8 is now affiliation 4:

^4^Department of Ophthalmology, Faculty of Medical Sciences, University of Fukui, Fukui, Japan

The original version of this article has been updated.

