# Supplementary material for: Salivary oxytocin responses to infant stimuli vary by EPDS scores among postpartum Japanese mothers without clinically diagnosed postpartum depression
Source: Front Endocrinol (Lausanne). 2025 Dec 17;16:1689899. doi: 10.3389/fendo.2025.1689899 (PMC12753409; doi:10.3389/fendo.2025.1689899)

Salivary oxytocin responses to infant stimuli vary by EPDS scores among postpartum Japanese mothers without clinically diagnosed postpartum

Kana Minami[1, 2], Haruhiro Higashida[1], Shigeru Yokoyama[1,3] , Takahiro Tsuji[1,4],

Naomi Kagami[2], Chiharu Tsuji[1,3] *

1. Research Center for Child Mental Development, Kanazawa University, Kanazawa, Japan.

2. Department of Health Development Nursing, Institute of Medical, Pharmaceutical and Health Sciences, Kanazawa University, Kanazawa, Japan.

3. Department of Socioneurosciences, United Graduate School of Child Development, Osaka University, Kanazawa University, Hamamatsu University School of Medicine, Chiba University and University of Fukui, Kanazawa Campus, Kanazawa, Japan.

4. Department of Ophthalmology, Faculty of Medical Sciences, University of Fukui, Fukui, Japan.

Suppl
Fig. 1

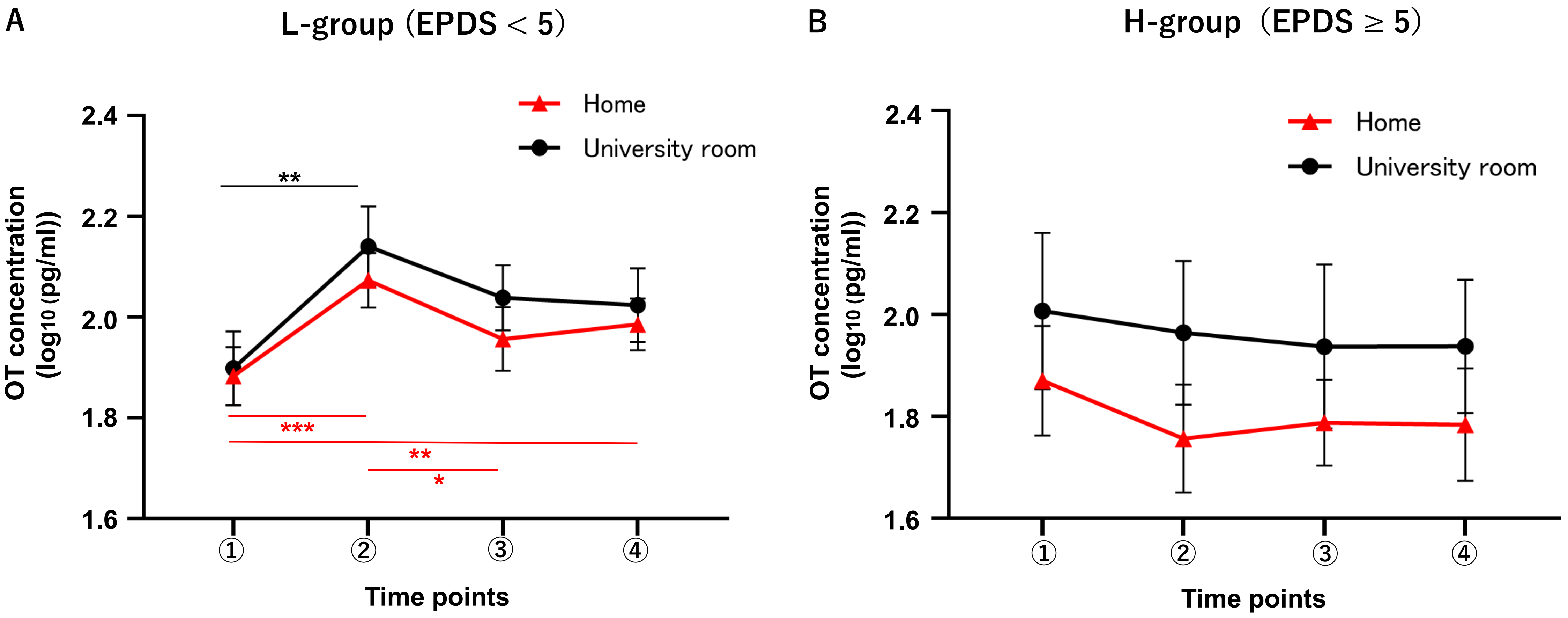

A
L-group（EPDS < 5）
Home
University room
OT concentration
(log10 (pg/ml))
2.4
2.2
2.0
1.8
1.6
**
***
**
*
①
②
③
④
Time points
B
H-group（EPDS ≥ 5）
Home
University room
OT concentration
(log10 (pg/ml))
2.4
2.2
2.0
1.8
1.6
①
②
③
④
Time points

Suppl
Fig. 2

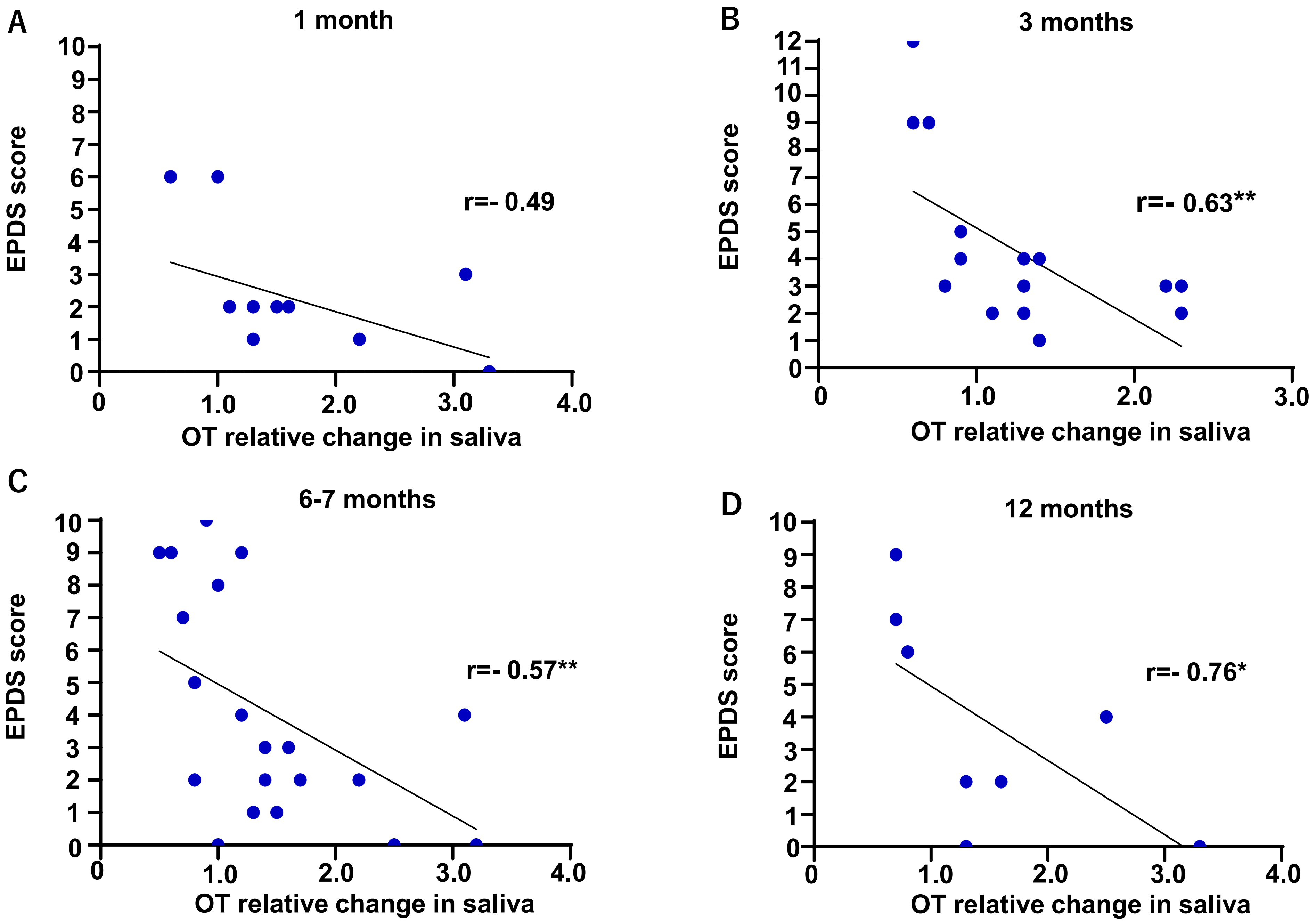
A
1 month
EPDS score
r=- 0.49
OT relative change in saliva
B
3 months
EPDS score
r=- 0.63**
OT relative change in saliva
C
6-7 months
EPDS score
r=- 0.57**
OT relative change in saliva
D
12 months
EPDS score
r=- 0.76*
OT relative change in saliva

Suppl
Fig. 3

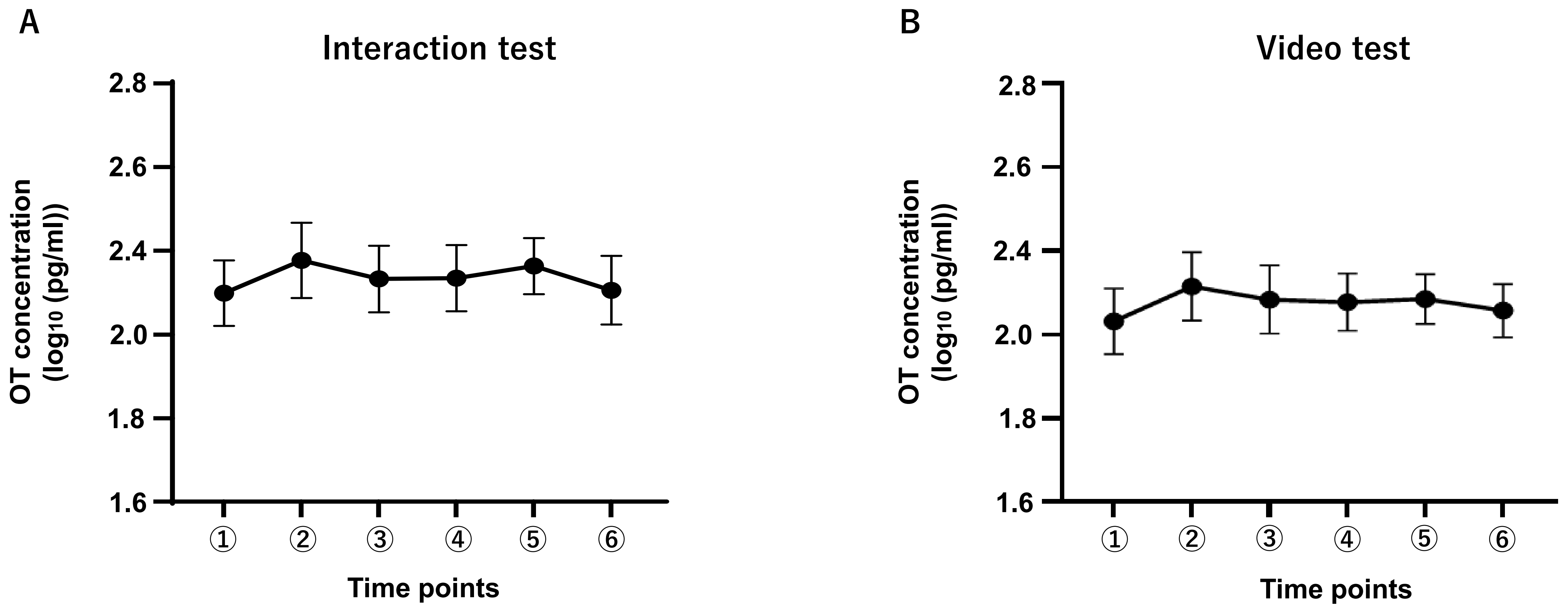
A
Interaction test
OT concentration (log10 (pg/ml))
2.8
2.6
2.4
2.0
1.8
1.6
① ② ③ ④ ⑤ ⑥
Time points
B
Video test
OT concentration (log10 (pg/ml))
2.8
2.6
2.4
2.0
1.8
1.6
① ② ③ ④ ⑤ ⑥
Time points

Suppl
Fig. 4

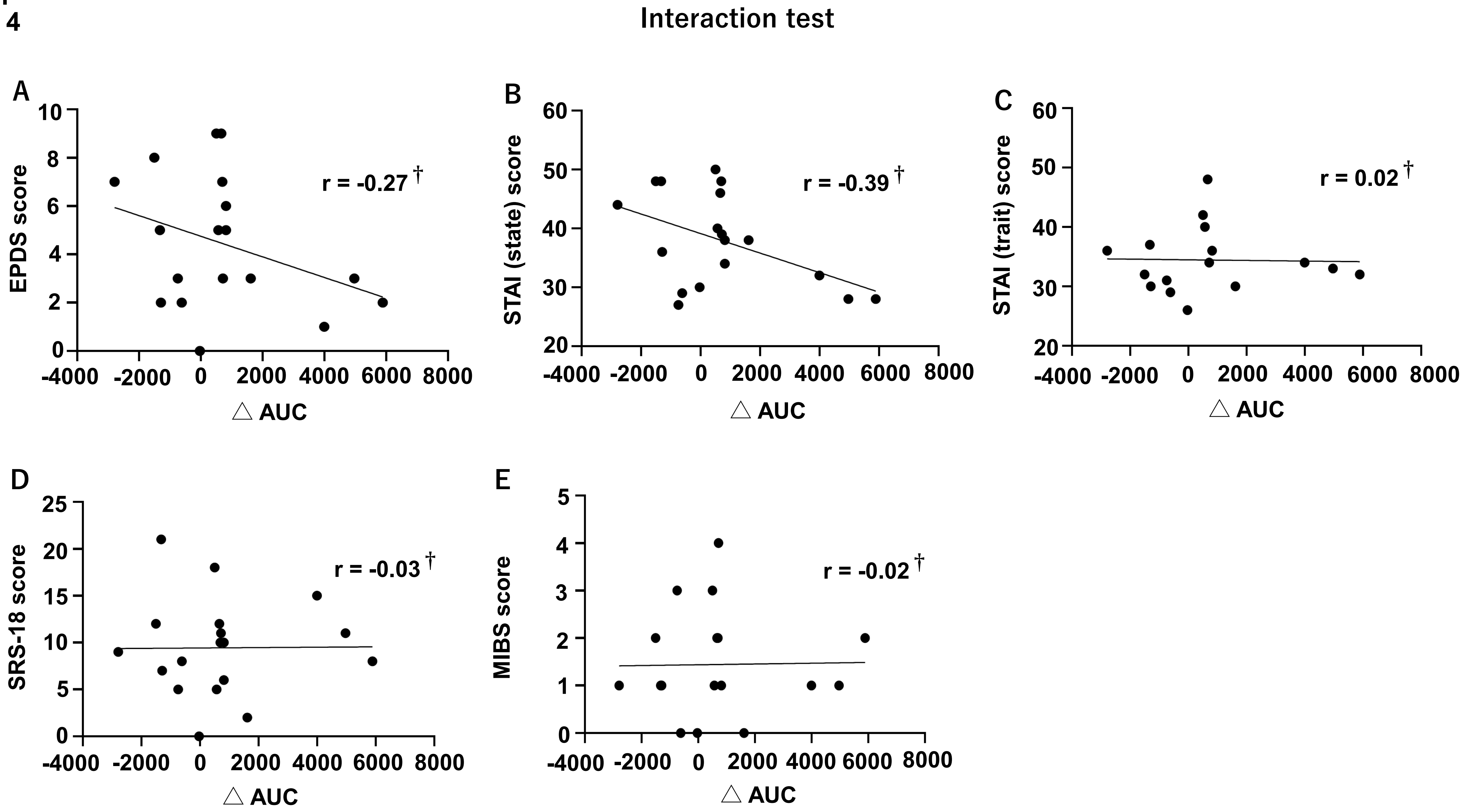

Interaction test
A
EPDS score
r = -0.27 †
△ AUC
B
STAI (state) score
r = -0.39 †
△ AUC
C
STAI (trait) score
r = 0.02 †
△ AUC
D
SRS-18 score
r = -0.03 †
△ AUC
E
MIBS score
r = -0.02 †
△ AUC

Suppl Fig. 5

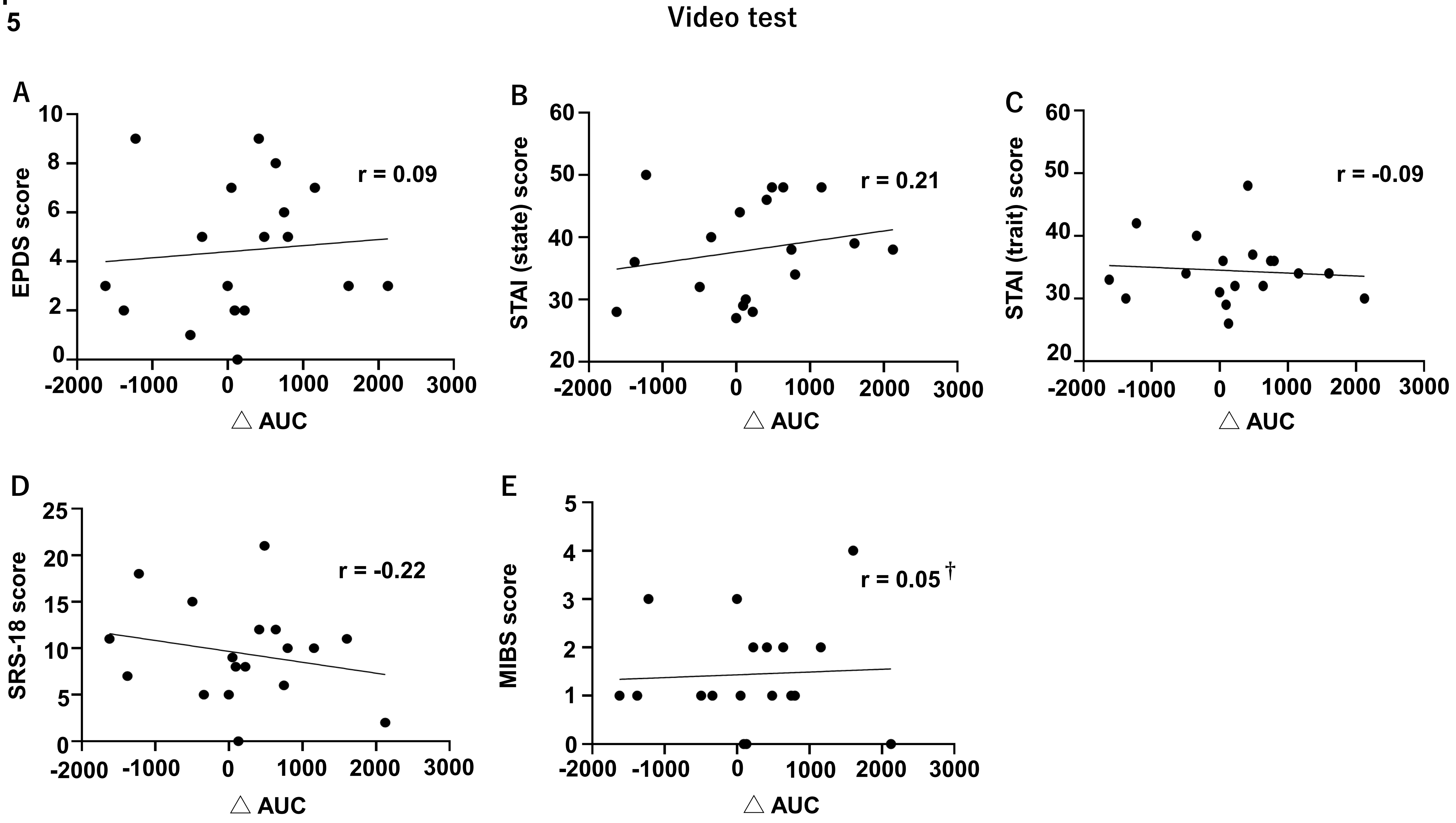

Video test
A
EPDS score
r = 0.09
10
8
6
4
2
0
-2000
-1000
0
1000
2000
3000
△ AUC
B
STAI (state) score
r = 0.21
60
50
40
30
20
C
STAI (trait) score
r = -0.09
D
SRS-18 score
r = -0.22
25
20
15
10
5
E
MIBS score
r = 0.05 †

Suppl Fig. 6

Interaction test

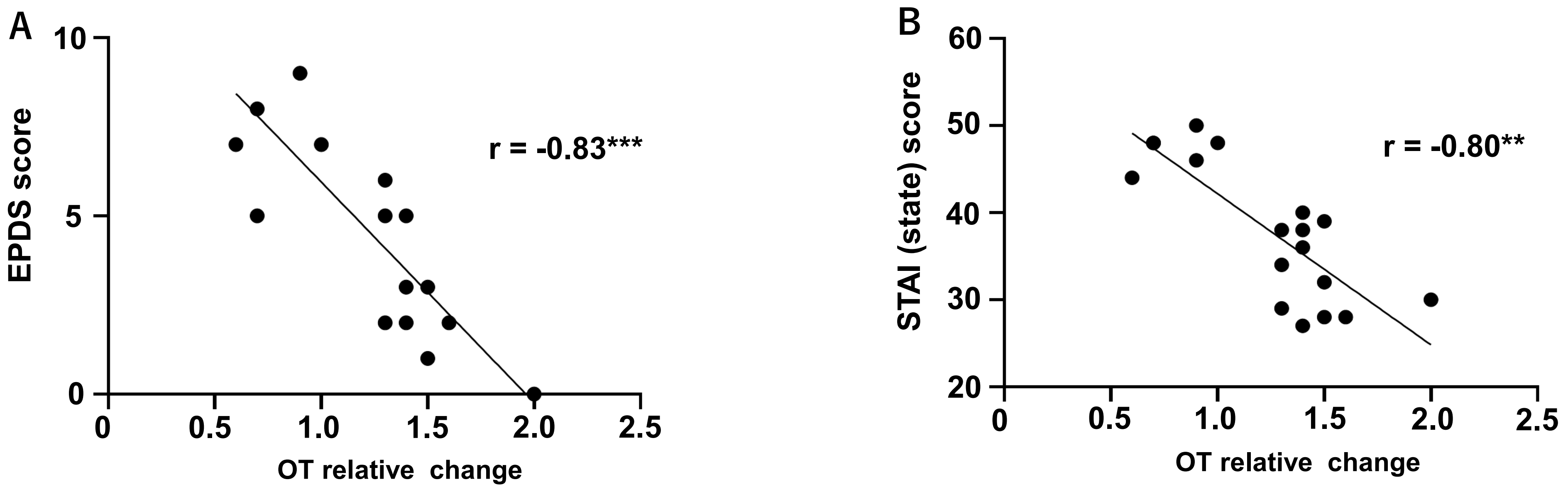

Video test

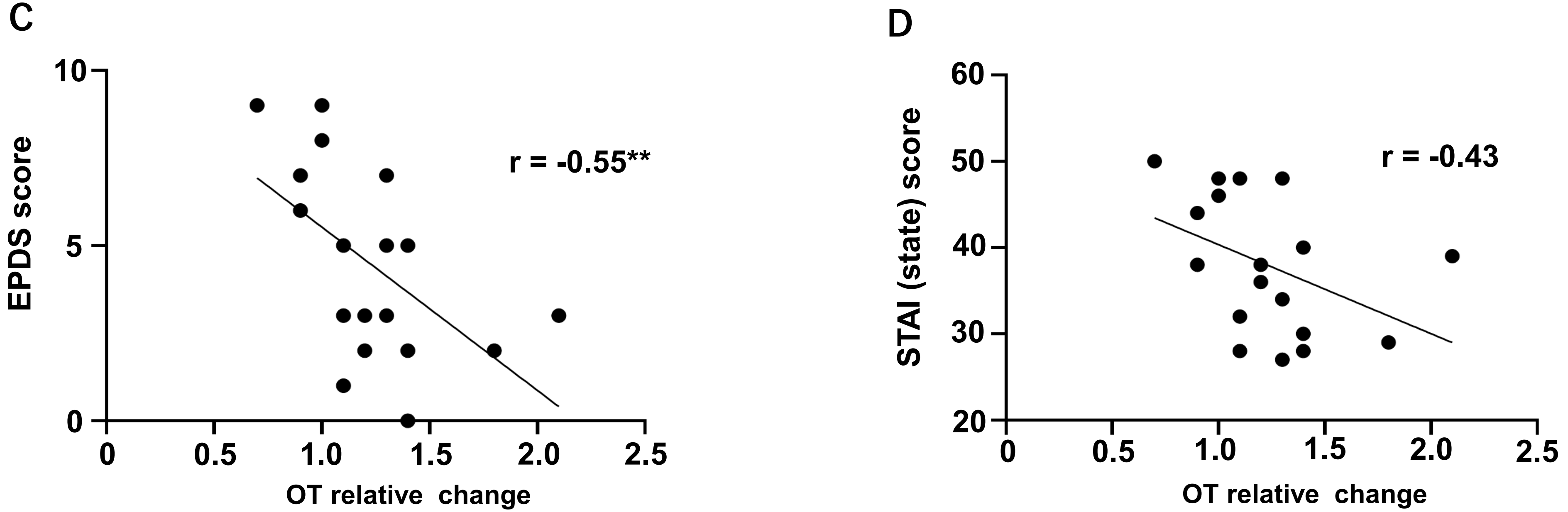

Supplement: Supplementary file 1 [file Image1.pdf]
