# Supplementary material for: Salivary oxytocin responses to infant stimuli vary by EPDS scores among postpartum Japanese mothers without clinically diagnosed postpartum depression
Source: Front Endocrinol (Lausanne). 2025 Dec 17;16:1689899. doi: 10.3389/fendo.2025.1689899 (PMC12753409; doi:10.3389/fendo.2025.1689899)
Supplement: Supplementary file 2 [file DataSheet1.pdf]

Salivary oxytocin responses to infant stimuli vary by EPDS scores among postpartum Japanese mothers without clinically diagnosed postpartum depression

Kana Minami[1, 2], Haruhiro Higashida[1], Shigeru Yokoyama[1,3], Takahiro Tsuji[1,4],

Naomi Kagami[2], Chiharu Tsuji[1,3 *]

1. Research Center for Child Mental Development, Kanazawa University, Kanazawa, Japan

2. Department of Health Development Nursing, Institute of Medical, Pharmaceutical and Health Sciences, Kanazawa University, Kanazawa, Japan.

3. Department of Socioneurosciences, United Graduate School of Child Development, Osaka University, Kanazawa University, Hamamatsu University School of Medicine, Chiba University and University of Fukui, Kanazawa Campus, Kanazawa, Japan

4. Department of Ophthalmology, Faculty of Medical Sciences, University of Fukui, Fukui, Japan.

Supplementary Figure 1

Changes in salivary OT concentration across different settings (home vs. university room).

(A) Changes in salivary OT concentration in the L-group, measured either at home (n = 32) or in a university room (n = 12). (B) Changes in salivary OT concentration in the H-group, measured either at home (n = 10) or in a university room (n = 7). Mean ± SEM. Within-group time points, *p < 0.05, **p < 0.01, ***p < 0.001.

Supplementary Figure 2

Correlation between relative change in salivary OT during breastfeeding and EPDS scores across postpartum periods.

Scatter plots showing the correlation between the relative change (RC; 5 min / basal) in salivary OT during the breastfeeding task and Edinburgh Postnatal Depression Scale (EPDS) scores, categorized by postpartum period:

(A) 1 month postpartum (n = 12)

(B) 3 months postpartum (n = 17)

(C) 6–7 months postpartum (n = 24)

(D) 12 months postpartum (n = 8)

Spearman’s correlation coefficients are shown. *p < 0.05, **p < 0.01.

Supplementary Figure 3

Changes in salivary OT concentration during the interaction test and video test.

(A) Changes in salivary OT concentration during the interaction test across all participants (n = 18). (B) Changes in salivary OT concentration during the video test across all participants (n = 18).

Supplementary Figure 4

Correlation between psychological scale scores and ΔAUC of salivary OT during interaction test. Scatter plots showing the correlation between ΔAUC (delta area under the curve) of salivary OT and psychological scale scores:

(A) Edinburgh Postnatal Depression Scale (EPDS),

(B) State subscale of the State-Trait Anxiety Inventory (STAI-State),

(C) Trait subscale of the State-Trait Anxiety Inventory (STAI-Trait),

(D) Stress Response Scale-18 (SRS-18),

(E) Mother-to-Infant Bonding Scale (MIBS).

Values are shown as Pearson’s correlation coefficients unless otherwise noted. †Spearman’s

rank correlation test.

Supplementary Figure 5

Correlation between psychological scale scores and ΔAUC of salivary OT during video test

Scatter plots showing the correlation between ΔAUC (delta area under the curve) of salivary OT and psychological scale scores:

(A) Edinburgh Postnatal Depression Scale (EPDS),

(B) State subscale of the State-Trait Anxiety Inventory (STAI-State),

(C) Trait subscale of the State-Trait Anxiety Inventory (STAI-Trait),

(D) Stress Response Scale-18 (SRS-18),

(E) Mother-to-Infant Bonding Scale (MIBS).

Values are shown as Pearson's correlation coefficients unless otherwise noted. †Spearman's rank correlation test.

Supplementary Figure 6

Correlation between relative change in salivary OT at 1 minute and psychological scale scores.

Scatter plots showing the correlation between the relative change (RC; 1 min/basal) in salivary OT during the interaction test (A, B) and the video test (C, D), plotted against:

(A, C) Edinburgh Postnatal Depression Scale (EPDS) score,

(B, D) State-Trait Anxiety Inventory (STAI) score.

Values are shown as Pearson's correlation coefficients. *$p < 0.05$, **$p < 0.01$.
