# Supplementary material for: Salivary oxytocin responses to infant stimuli vary by EPDS scores among postpartum Japanese mothers without clinically diagnosed postpartum depression
Source: Front Endocrinol (Lausanne). 2025 Dec 17;16:1689899. doi: 10.3389/fendo.2025.1689899 (PMC12753409; doi:10.3389/fendo.2025.1689899)
Supplement: Supplementary file 3 [file Table1.pdf]

Supplementary Table 1. **Correlation between the △AUC and psychological scale scores**

	Psychological scale scores				
	EPDS	STAI state	STAI trait	SRS-18	MIBS†
△ AUC during interaction test†	-0.27	-0.39	0.02	-0.03	-0.02
△ AUC during video test	0.09	0.21	-0.09	-0.22	0.05

Pearson’s correlation coefficients unless noted. † Spearman’s rank correlation test.
